# Cost-Effectiveness of Risk-Stratified Colorectal Cancer Screening Based on Polygenic Risk: Current Status and Future Potential

**DOI:** 10.1093/jncics/pkz086

**Published:** 2019-10-14

**Authors:** Steffie K Naber, Suman Kundu, Karen M Kuntz, W David Dotson, Marc S Williams, Ann G Zauber, Ned Calonge, Doris T Zallen, Theodore G Ganiats, Elizabeth M Webber, Katrina A B Goddard, Nora B Henrikson, Marjolein van Ballegooijen, A Cecile J W Janssens, Iris Lansdorp-Vogelaar

**Affiliations:** 1 Department of Public Health, Erasmus MC, University Medical Center Rotterdam, Rotterdam, the Netherlands; 2 Division of Cardiovascular Medicine, Vanderbilt University Medical Center, Nashville, TN; 3 Division of Health Policy & Management, University of Minnesota School of Public Health, Minneapolis, MN; 4 Office of Public Health Genomics, Centers for Disease Control and Prevention, Atlanta, GA; 5 Genomic Medicine Institute, Geisinger, Danville, PA; 6 Memorial Sloan Kettering Cancer Center, New York, NY; 7 The Colorado Trust, Denver, CO; 8 Department of Science, Technology, and Society, Virginia Tech, Blacksburg, VA; 9 Department of Basic Science Education, Virginia Tech-Carilion School of Medicine, Roanoke, VA; 10 Department of Family Medicine and Public Health, University of California San Diego, La Jolla, CA; 11 Center for Health Research, Kaiser Permanente, Portland, OR; 12 Kaiser Permanente Washington Health Research Institute, Seattle, WA; 13 Department of Clinical Genetics, EMGO Institute for Health and Care Research, Section Community Genetics, VU University Medical Center, Amsterdam, the Netherlands; 14 Department of Epidemiology, Rollins School of Public Health, Emory University, Atlanta, GA

## Abstract

**Background:**

Although uniform colonoscopy screening reduces colorectal cancer (CRC) mortality, risk-based screening may be more efficient. We investigated whether CRC screening based on polygenic risk is a cost-effective alternative to current uniform screening, and if not, under what conditions it would be.

**Methods:**

The MISCAN-Colon model was used to simulate a hypothetical cohort of US 40-year-olds. Uniform screening was modeled as colonoscopy screening at ages 50, 60, and 70 years. For risk-stratified screening, individuals underwent polygenic testing with current and potential future discriminatory performance (area under the receiver-operating curve [AUC] of 0.60 and 0.65–0.80, respectively). Polygenic testing results were used to create risk groups, for which colonoscopy screening was optimized by varying the start age (40–60 years), end age (70–85 years), and interval (1–20 years).

**Results:**

With current discriminatory performance, optimal screening ranged from once-only colonoscopy at age 60 years for the lowest-risk group to six colonoscopies at ages 40–80 years for the highest-risk group. While maintaining the same health benefits, risk-stratified screening increased costs by $59 per person. Risk-stratified screening could become cost-effective if the AUC value would increase beyond 0.65, the price per polygenic test would drop to less than $141, or risk-stratified screening would lead to a 5% increase in screening participation.

**Conclusions:**

Currently, CRC screening based on polygenic risk is unlikely to be cost-effective compared with uniform screening. This is expected to change with a greater than 0.05 increase in AUC value, a greater than 30% reduction in polygenic testing costs, or a greater than 5% increase in adherence with screening.

Colorectal cancer (CRC) is the second most common cause of cancer death in the United States, with more than 51 000 deaths expected in 2019 (1). Fortunately, screening and early treatment of adenomas and CRC can prevent CRC death (2). Randomized, controlled trials have shown that CRC mortality can be reduced by 15–30% with fecal occult blood testing, and by 40% with flexible sigmoidoscopy screening (3–5). Colonoscopy screening is expected to achieve an even higher mortality reduction (6). 

Of US adults who were up to date with screening in 2012, two-thirds were screened by colonoscopy (7). Because this is an invasive procedure, with the potential for serious complications (8), it could be argued that those who would not have been diagnosed with CRC in the absence of screening (approximately 95% of the population) face unnecessary risks of colonoscopy screening. Meanwhile, others may have had a better prognosis if screening would have detected their disease at an earlier time. If screening could be more targeted, then mortality could be reduced in those at increased CRC risk, while harms could be reduced in those at decreased CRC risk. To identify those at increased or decreased risk, exploratory studies have suggested the use of polygenic risk profiling (9, 10).

Polygenic risk prediction differs from commonly described hereditary CRC syndromes by its focus on multiple single nucleotide polymorphisms (SNPs) instead of a single genetic mutation. For individuals with identified inherited syndromes that are caused by a single genetic mutation, such as Lynch syndrome, more intensive screening is already recommended (11). The 2–5% of CRC cases that can be attributed to high-penetrance genes causing these types of syndromes account for only a small fraction of the total heritability of CRC, which has been estimated at 12–35% (12,13). A comprehensive whole-genome sequencing study and meta-analysis of genome-wide association studies recently identified 40 new independent signals for CRC, bringing the number of known independent signals for CRC to about 100 (14). Together, these signals explain approximately 11% of the familial relative risk in US individuals (14). As more genetic variants associated with CRC risk are detected, this percentage could potentially increase to 73% (15). Another study suggested that if all variants were identified, at least 7.42% of all CRC cases would be explained by SNPs (16).

The benefits of screening based on polygenic risk will depend on the discriminatory accuracy of risk-stratification algorithms in identifying those who will develop CRC, as expressed by the area under the receiver-operating characteristic curve (AUC). With the currently identified common genetic variants, the AUC value of polygenic testing is approximately 0.6 (17,18). As more variants are discovered, this value may further increase. In this study, we estimated whether risk-stratified screening would be a cost-effective alternative to current uniform screening, and if not, under what conditions it would be.

## Methods

We used the Microsimulation Screening Analysis-Colon (MISCAN-Colon) model to simulate a population at average risk for CRC that is willing to undergo polygenic testing and subsequent risk-stratified colonoscopy screening. We compared the results of such risk-stratified screening with those of current uniform screening, with colonoscopies at ages 50, 60, and 70 years (19).

### MISCAN-Colon

MISCAN-Colon is a well-established microsimulation model for CRC developed at the Department of Public Health of the Erasmus University Medical Center (Rotterdam, the Netherlands) (20). The structure, underlying assumptions, and calibration of the model are described in previous publications and in the Model Appendix (available online) (20,21). In brief, MISCAN-Colon simulates the life histories of a large population from birth until death. As each simulated person ages, one or more adenomas may develop. These adenomas can progress from small (≤5 mm), to medium (6–9 mm), to large size (≥10 mm). Some adenomas can develop into preclinical cancer, which may progress through stages I to IV. During each stage CRC may be diagnosed because of symptoms. Survival after clinical diagnosis is determined according to the stage at diagnosis, the localization of the cancer, and the person’s age (22).

Screening will alter some simulated life histories through cancer prevented by adenoma detection and removal, or cancers detected at earlier stages resulting in more favorable survival estimates. However, screening can also result in serious complications, as well as overdiagnosis and overtreatment of CRC (ie, the detection and treatment of cancers that would not have been detected without screening). By comparing a simulation of life histories with screening to a simulation of the same life histories without screening, MISCAN-Colon quantifies the effectiveness of screening, as well as the associated harms and costs.

### Simulated Population in MISCAN-Colon

We simulated a cohort of 40-year-olds with US life expectancy and followed them until death (23). At model initiation, each individual is assigned an underlying baseline risk of developing adenomas. An adenoma can be either progressive (ie, may cause cancer dependent on the duration) or nonprogressive (ie, will never cause cancer). Once a progressive adenoma has developed, the duration until cancer development is randomly drawn, and it does not depend on any individual-based risk level.

### Polygenic Testing

By testing for the presence of risk alleles that are associated with CRC, a polygenic test can estimate someone’s relative risk (RR) of developing CRC. The larger the number of risk alleles included in such a test, the more accurate its estimate of someone’s risk, and the higher its AUC value. A previously published method was used to generate the RR distribution in hypothetical populations with varying AUC values of polygenic testing of 0.60, 0.65, 0.70, 0.75, and 0.80 (Supplementary AppendixFigure 1 available online) (24,25). We split the simulated population into groups of estimated RR for cancer, using an elliptical copula in R. This method assigned an individual to an RR group as a function of underlying baseline risk and discriminative accuracy of the polygenic test: The higher the AUC value, the higher the correlation of estimated RR and underlying baseline risk (see Technical Appendix available online for details). For risk-stratified screening, each population was split into RR groups, with RR varying from less than 0.1 to 0.1 to 5.9 with increments of 0.1 to greater than 5.9, yielding 60 groups.

**Figure 1.**
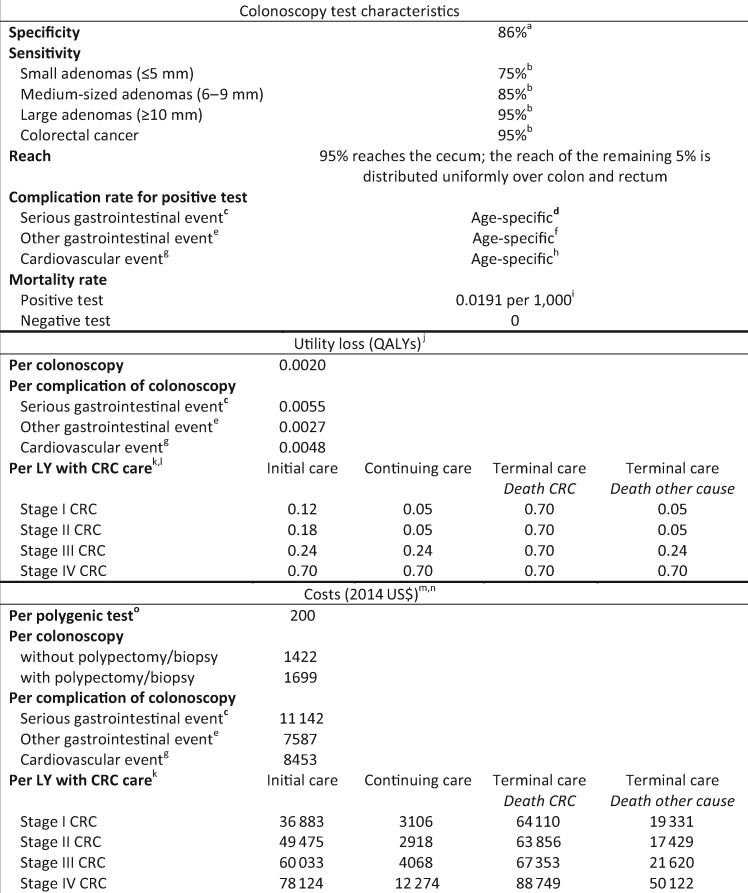
Model inputs: test characteristics, utility loss, and costs of colorectal cancer screening and treatment. **a**) We assumed that in 14% of all negative colonoscopies a nonadenomatous lesion was detected, resulting in a polypectomy or a biopsy, respectively. **b**) The sensitivity of colonoscopy for the detection of adenomas and CRC within the reach of the endoscope was obtained from a systematic review on miss rates observed in tandem colonoscopy studies (26). **c**) Serious gastrointestinal events are perforations, gastrointestinal bleeding, or transfusions. **d**) Formula: 1/[exp(9.27953 − 0.06105 × Age) + 1] − 1/[exp(10.78719 − 0.06105 × Age) + 1]. **e**) Other gastrointestinal events are paralytic ileus, nausea and vomiting, dehydration, or abdominal pain. **f**) Formula: 1/[exp(8.81404 − 0.05903 × Age) + 1] − 1/[exp(9.61197 − 0.05903 × Age) + 1]. **g**) Cardiovascular events are myocardial infarction or angina, arrhythmias, congestive heart failure, cardiac or respiratory arrest, syncope, hypotension, or shock. **h**) Formula: 1/[exp(9.09053 − 0.07056 × Age) + 1] − 1/[exp(9.38297 − 0.07056 × Age) + 1]. **i**) Risk of dying from a colonoscopy at age 65 (Warren et al. [27], Gatto et al. [28], and van Hees et al. [29]) **j**) The loss of quality of life associated with a particular event. **k**) Care for CRC was divided in three clinically relevant phases: initial, continuing, and terminal care. The initial care phase was defined as the first 12 months after diagnosis; the terminal care phase was defined as the final 12 months of life; the continuing care phase was defined as all months in between. In the terminal care phase, we distinguished between CRC patients dying from CRC and CRC patients dying from another cause. For patients surviving less than 24 months, the final 12 months were allocated to the terminal care phase, and the remaining months were allocated to the initial care phase. **l**) Utility losses for LYs with initial care were derived from a study by Ness and colleagues (30). For LYs with continuing care for stage I and II CRC, we assumed a utility loss of 0.05 QALYs; for LYs with continuing care for stage III and IV CRC, we assumed the corresponding utility losses for LYs with initial care. For LYs with terminal care for CRC, we assumed the utility loss for LYs with initial care for stage IV CRC. For LYs with terminal care for another cause, we assumed the corresponding utility losses for LYs with continuing care. **m**) Costs include copayments and patient time costs (ie, the opportunity costs of spending time on screening or being treated for a complication or CRC), but do not include travel costs, costs of lost productivity, and unrelated health-care and non–health-care costs in added years of life. We assumed that the value of patient time was equal to the median wage rate in 2014: $17.09 per hour (31). We assumed that colonoscopies used up 36 hours; serious gastrointestinal complications 192 hours; other gastrointestinal complications 96 hours; and cardiovascular complications 120 hours of patient time. Patient-time costs associated with CRC care were provided by Yabroff (personal communication) and were calculated using the methodology described in a study by Yabroff and colleagues (32). **n**) In sensitivity analyses, all costs except those of polygenic testing were increased to reflect commercial rates rather than Medicare reimbursement; see Supplementary AppendixTable 1 (available online) (33). **o**) Polygenic testing costs for CRC risk were based on the price of a currently available polygenic test (34). Alternative values ($100 and $300) were considered in sensitivity analyses. QALY = quality-adjusted life-year; LY = life-year; CRC = colorectal cancer.

### Screening and Surveillance

We simulated scenarios without screening and with colonoscopy screening at intervals of 1, 2, 3, 5, 7, 10, 15, and 20 years. In addition, different begin ages (40, 45, 50, 55, and 60 years) and different end ages (70, 75, 80, and 85 years) for screening were considered. Test characteristics of colonoscopy are shown in Figure 1.

Individuals with adenomas detected and removed at screening were assumed to undergo colonoscopy surveillance according to current guidelines (35). Because surveillance is meant to follow individuals at increased risk more closely, the recommended surveillance interval was shortened for strategies for which it would have been longer than the screening interval. We assumed that surveillance continued until 5 years after the end age of screening.

### Adherence

In the base-case analysis, we assumed full adherence to polygenic testing, colonoscopy screening, and colonoscopy surveillance because we aim to estimate screening outcomes for those willing to undergo screening. In sensitivity analyses, we assumed percentages that are more realistic, based on observed adherence rates in the United States (36).

### Costs and Utilities

The assumed loss in quality of life attributed to CRC screening was 0.002 quality-adjusted life-years (QALYs) per colonoscopy (1.5 days at 0.5 utility) and 0.0027–0.0055 QALYs per complication of colonoscopy (2–4 days at 0.5 utility) (Figure 1). We also assumed that life-years (LYs) with CRC are of lower quality than those without CRC (30).

The cost-effectiveness analyses were conducted from a modified societal perspective. We included both direct medical costs, as well as time costs both for the patient and a patient escort. However, direct non–health-care costs such as travel expenses were not included (37). The costs of colonoscopies were based on 2014 Medicare payment rates and copayments (Figure 1). For complications, the average payment by Centers for Medicare and Medicaid Services (CMS) was calculated by type of complication using frequency data on hospitalizations for colonoscopy complications from Craig Parzynski, MS, of Yale University (personal communication). Net costs of CRC care were obtained from an analysis of Surveillance, Epidemiology, and End Results–Medicare–linked data (38) (personal communication, Robin Yabroff, PhD, and Martin Brown, PhD, both formerly of the National Cancer Institute). Patient-time costs and copayments were added to all these estimates, which were then updated to 2014 US dollars using the Consumer Price Index (39). For polygenic testing, we assumed a cost of $200 based on the price of currently available polygenic tests (34).

### Analyses and Outcomes

For every level of underlying baseline risk, we simulated all screening strategies to quantify their QALYs and costs as compared with no screening. For uniform screening, we simply calculated a weighted sum of the results of colonoscopy screening at ages 50, 60, and 70 years over all underlying risk groups. For risk-stratified screening, we first calculated QALYs and costs by *estimated* RR group for every screening strategy as a weighted average of the outcomes by underlying risk within that *estimated* RR group. Next, we used these results to determine the optimal screening strategy by *estimated* RR group. Finally, the costs and effects of all optimal strategies were summed to obtain overall outcomes for risk-stratified screening (see Technical Appendix available online for details).

For risk-stratified screening, the willingness-to-pay (WTP) threshold was set to the level at which the QALYs gained were equal to those of uniform screening. This enabled a fair comparison of the costs of uniform vs risk-stratified screening. In the results we show the costs of both strategies, as well as the cost savings when uniform screening would be replaced with risk-stratified screening. We applied the conventional 3% annual discount rate both for costs and effects.

### Sensitivity Analyses

In one-way sensitivity analyses, we varied:

the costs of polygenic testing – assuming a price per test of $100 and $300 rather than $200; the costs of CRC screening, complications and treatment – to account for commercial rates at less than age 65, we multiplied health-care costs of CRC screening, complications, and treatment with a ratio of 1.35, as suggested by Ladabaum et al. (33); the complexity of risk stratification – by including a maximum of three instead of 60 risk groups, where the 60 risk groups were merged into three risk groups that were as equal in size as possible, or by assuming that every risk group would receive at least one screening colonoscopy; and the adherence with polygenic testing and subsequent screening – by assuming a more realistic uptake of 60% for uniform screening (36), and of 54%, 57%, 60%, 63%, and 66% for risk-stratified screening. For an equal uptake of 60%, we also varied the percentage of the population that undergoes polygenic testing but does not participate in CRC screening from 0% (base case) to 5% and 10%.

## Results

**Figure 2.**
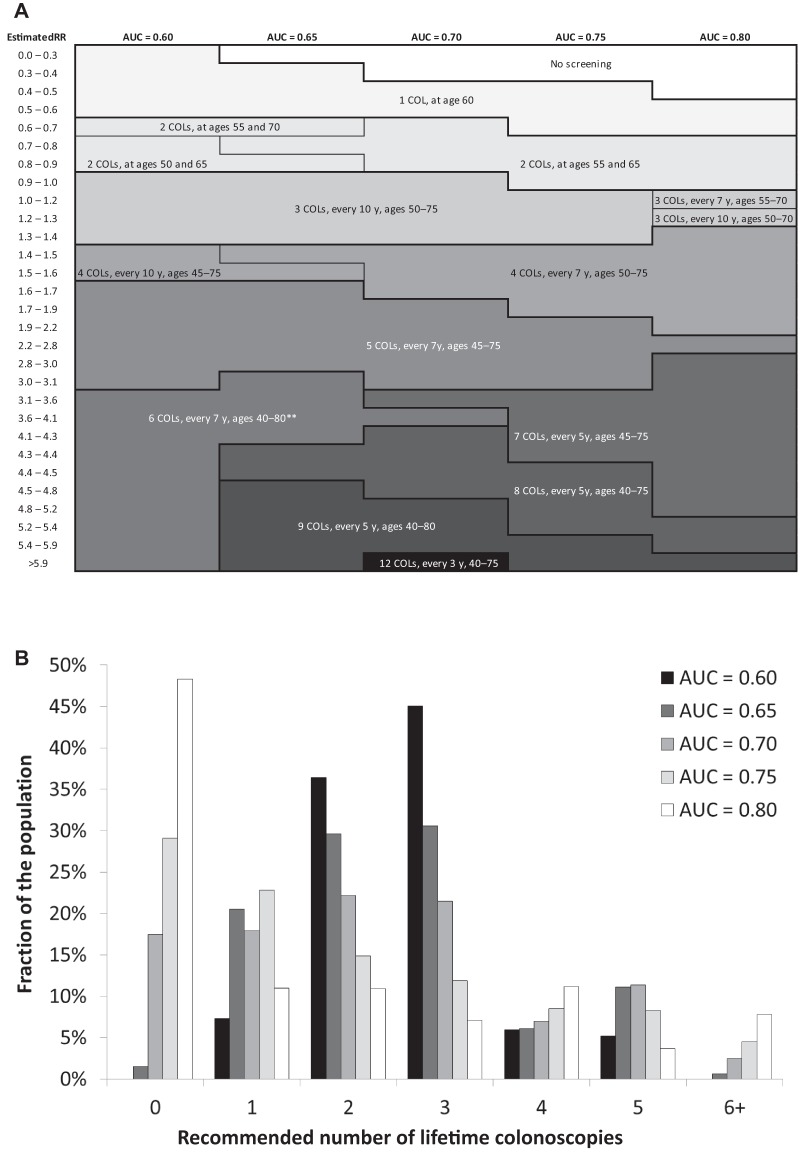
**A**) Risk-stratified screening strategies by relative risk (RR) as estimated by a polygenic test with AUC value of 0.60–0.80, given a willingness-to-pay threshold for risk-stratified screening that ensures that the entire risk-stratified screening program yields as least as many QALYs as a uniform screening program with colonoscopies at ages 50, 60, and 70 years.* For every strategy, the number of lifetime colonoscopies, screening interval, and age range of screening is given (ie, “3 COLs, every 10 y, ages 50–75” refers to three lifetime colonoscopies with an interval of 10 years in individuals aged 50–75 years). **B**) Distribution of recommended numbers of lifetime colonoscopies in the population for different AUC values. RR = relative risk; AUC = area under the receiver-operating characteristic curve; COLs = colonoscopies. *Willingness-to-pay threshold equals $69 000, $65 000, $56 700, $46 000, and $38 500 for AUC = 0.60, 0.65, 0.70, 0.75, and 0.80, respectively. **For AUC = 0.70, individuals with an estimated RR of 3.6–4.1 are offered fewer lifetime screens than those with an estimated RR of 3.1–3.6, but the age range in which they are offered screening is broader.

Compared with no screening, uniform screening yielded 73 LYs and 85 QALYs per 1000 40-year-olds, at a total cost of $1 626 000. At a WTP threshold of $69 000 per QALY, risk-stratified screening with current discriminatory performance of polygenic testing resulted in an equal number of QALYs (Table 1). Optimal screening strategies ranged from one colonoscopy at age 60 years for the lowest-risk group (ie, those with an estimated RR less than 0.6) to six colonoscopies at ages 40–80 years for the highest-risk group (ie, those with an estimated RR greater than 3.1) (Figure 2A). However, more than 80% of individuals would be offered two or three lifetime colonoscopies (Figure 2B). A risk-stratified screening program including those strategies would require 131 fewer colonoscopies per 1000 40-year-olds than uniform screening yielding the same number of QALYs. Although CRC screening and treatment costs would also be $141 000 lower, the addition of polygenic testing costs implies that total costs of risk-stratified screening were $59 000 higher than those of uniform screening, suggesting that risk-stratified screening would not be considered cost-effective. However, not surprisingly, if polygenic testing cost $100 rather than $200 per person, then risk-stratified screening would be considered cost-effective. When assuming health-care costs based on commercial rates rather than Medicare reimbursement, CRC screening and treatment costs were higher both for uniform and risk-stratified screening (Supplementary AppendixTable 1 available online). The cost difference would then equal $168 000, suggesting that risk-stratified screening could be considered cost-effective if polygenic testing costs were less than $168 per person.

**Table 1.** Effects and costs per 1000 40-year-old individuals for no screening, uniform screening with colonoscopy at ages 50, 60, and 70 years, and risk-stratified screening, given a willingness-to-pay threshold for risk-stratified screening that ensures that the entire risk-stratified screening program yields as least as many QALYs as a uniform screening program with colonoscopies at ages 50, 60, and 70 years*

Strategy	Colonoscopies†		CRC cases		CRC deaths		Life-years‡		QALYs‡		Costs, USD (*1000)‡,§							
											Polygenic testing		CRC screening‖		Cancer diagnosis and treatment		Total	
No screening	67		67		28		22 940		22 907		0		4		2477		2481	
Uniform screening¶	3247	(referent)	30	(referent)	8	(referent)	23 014	(referent)	22 993	(referent)	0	(referent)	2809	(referent)	1298	(referent)	4107	(referent)
Risk-stratified screening#																		
Base case**	3116	(−131)	28	(−1)	8	(−1)	23 013	(−0)	22 993	(+0)	200	(+200)	2678	(−131)	1289	(−10)	4166	(+59)
Alternative costs of polygenic testing																		
$100 per person	3116	(−131)	28	(−1)	8	(−1)	23 013	(−0)	22 993	(+0)	100	(+100)	2678	(−131)	1289	(−10)	4166	(−41)
$300 per person	3116	(−131)	28	(−1)	8	(−1)	23 013	(−0)	22 993	(+0)	300	(+300)	2678	(−131)	1289	(−10)	4166	(+159)
Alternative discriminatory performance of polygenic testing																		
AUC = 0.65	2954	(−292)	29	(−1)	8	(−0)	23 013	(−0)	22 993	(+0)	200	(+200)	2529	(−280)	1287	(−11)	4017	(−91)
AUC = 0.70	2615	(−632)	29	(−0)	8	(+0)	23 013	(−1)	22 993	(+0)	200	(+200)	2255	(−553)	1289	(−10)	3744	(−363)
AUC = 0.75	2273	(−973)	30	(+0)	8	(+0)	23 012	(−1)	22 993	(+0)	200	(+200)	1967	(−841)	1295	(−4)	3462	(−645)
AUC = 0.80	1952	(−1294)	30	(+0)	9	(−1)	23 012	(−2)	22 993	(+0)	200	(+200)	1700	(−1108)	1295	(−3)	3196	(−912)

AUC = area under the receiver-operating characteristic curve; CRC = colorectal cancer; QALYs = quality-adjusted life-years; Referent = reference value; (n) = increase/decrease compared with uniform screening.

* Willingness-to-pay threshold equals $69 000, $65 000, $56 700, $46 000, and $38 500 for AUC = 0.60, 0.65, 0.70, 0.75, and 0.80, respectively.

† Includes screening colonoscopies, surveillance colonoscopies, and diagnostic colonoscopies.

‡ (Quality-adjusted) life-years and costs were discounted at an annual rate of 3%.

§ Costs are in 2014 US dollars (USD).

‖ Includes costs of screening colonoscopies, surveillance colonoscopies, and colonoscopy complications.

¶ Uniform screening was defined as colonoscopy screening at ages 50, 60, and 70 years for all.

# Figure 2 provides an overview of the screening strategies by relative risk for each AUC level.

** Base-case AUC value of polygenic testing is 0.60; base-case cost of polygenic testing is $200 per person.

**Figure 3.**
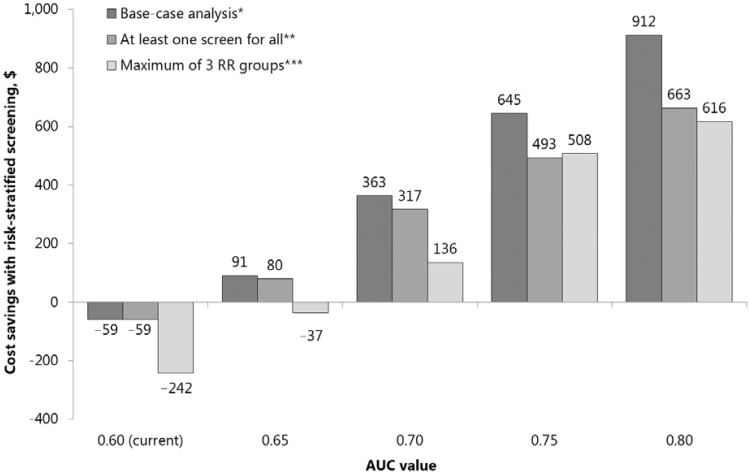
Cost savings of replacing uniform screening with risk-stratified screening, when risk-stratified screening yields (at least) as many QALYs as uniform screening, for the base-case analysis and for sensitivity analyses on the assumed complexity of risk-stratified screening. AUC = area under the receiver-operating characteristic curve; QALY = quality-adjusted life-year; RR = relative risk. *For screening strategies by RR group, see Figure 2. **For screening strategies by RR group, see Supplementary AppendixFigure 2 (available online). *** Individuals were grouped so that RR groups were as equal in size as possible. For screening strategies by RR group, see Supplementary AppendixFigure 3 (available online).

With greater discriminatory performance of polygenic testing, colonoscopies were more targeted to those in need (Figure 2, A and B). With an increase in AUC value from 0.60 to 0.80, the fraction of the population offered six or more lifetime colonoscopies increased from 0% to 12%, and the fraction in which screening could be forgone increased from 0% to 48%. This re-allocation of colonoscopies was associated with a reduced overall colonoscopy demand (Table 1) and made risk-stratified screening cost saving compared to uniform screening (Figure 3). Based on cost-effectiveness, risk-stratified screening would therefore be preferred.

### Sensitivity Analyses

Restricting the number of risk groups to three decreased the potential benefit of risk-stratified screening and thus the cost savings (Figure 3). Risk-stratified screening would be cost saving only if the AUC value of polygenic testing were to increase beyond 0.65. Offering at least one colonoscopy did not change the results for AUC = 0.60 because all risk groups were already offered one or more colonoscopies in the base-case analysis. For higher AUC values, a minimum of one colonoscopy did result in lower cost savings.

The additional cost of polygenic testing could be compensated for by a gain in screening participation. At current discriminatory performance, a 5% increase would already make risk-stratified screening less costly compared with uniform screening (Figure 4A). However, if switching to risk-stratified screening would imply a decrease in screening adherence of at least 5%, then risk-stratified screening would not be cost saving, even if polygenic testing were free.

**Figure 4.**
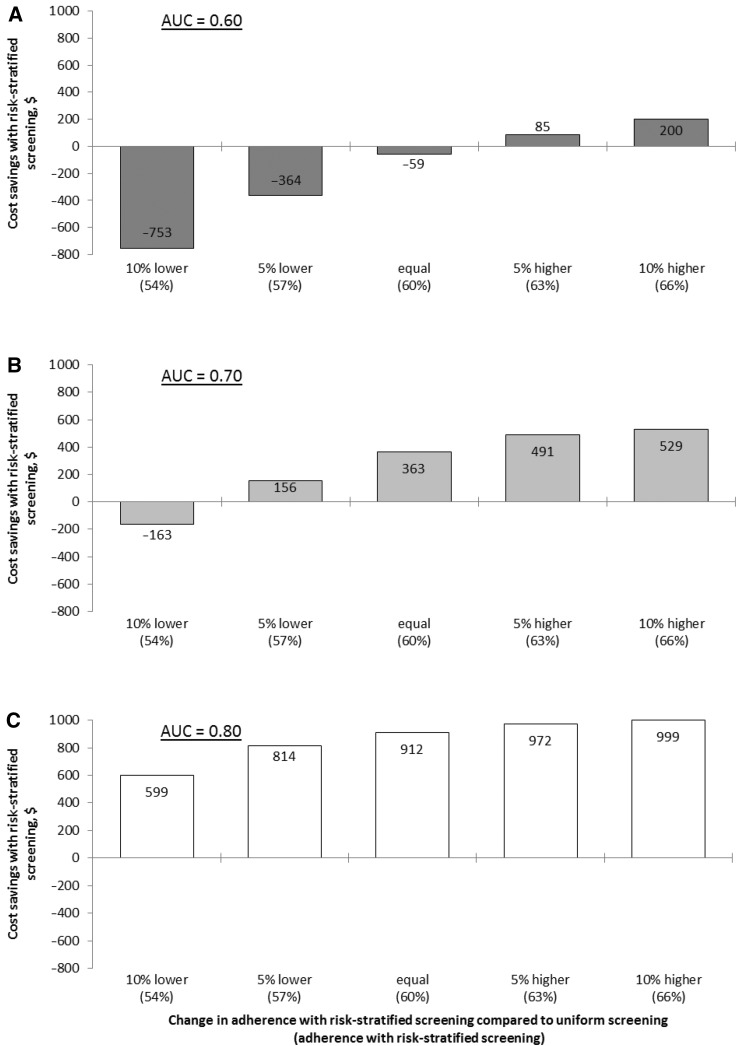
**A–C**) Estimated cost savings of replacing uniform screening with risk-stratified screening, when risk-stratified screening yields (at least) as many QALYs as uniform screening, for different levels of adherence with risk-stratified screening. **D–F**) Estimated cost savings of replacing uniform screening with risk-stratified screening, when risk-stratified screening yields (at least) as many QALYs as uniform screening, for different percentages of the population that does take polygenic testing but does not participate in CRC screening. In these panels, an adherence of 60% was assumed both for uniform and risk-stratified screening.

**Figure 4.**
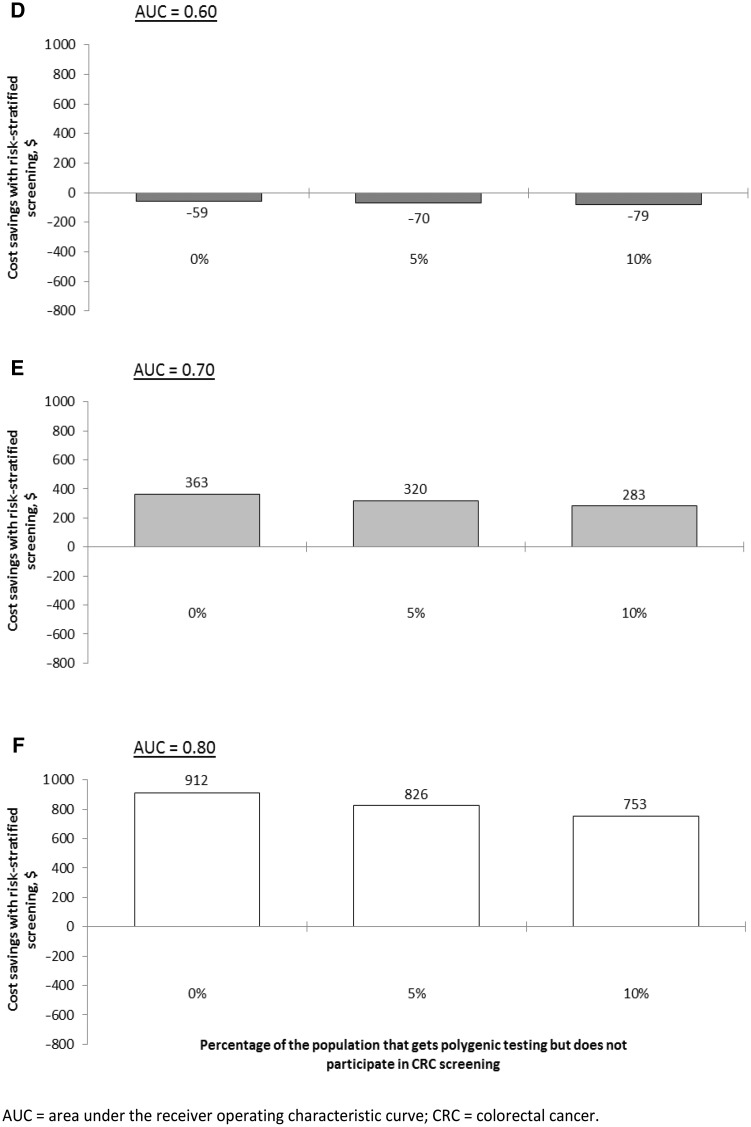
Continued.

With improved risk stratification, cost savings were less affected by potential changes in screening participation (Figure 3, B and C). Screening adherence could decrease with 5% (AUC = 0.70) or 10% (AUC = 0.80), and risk-stratified screening would still be more efficient than uniform screening.

If part of the population were to undergo polygenic testing but not attend CRC screening, costs of risk-stratified screening would increase, but its overall cost-effectiveness compared with uniform screening was relatively insensitive to this parameter (Figure 3, D–F).

## Discussion

This study shows that, under current discriminatory performance of polygenic testing (ie, AUC = 0.60), the health benefits of risk-stratified screening are modest at most. At a current estimated price of $200 per polygenic test, risk-stratified screening is not cost-effective compared with uniform screening. This could change if the discovery of more common genetic variants were to increase the AUC value of polygenic testing beyond 0.65. Alternatively, risk-stratified screening could become cost-effective at a lower price per polygenic test, or if adherence with risk-stratified screening were higher than with current uniform screening.

We estimated that from an AUC value of 0.65 onward, the reduction in CRC screening and treatment costs was sufficient to cover the expected costs of polygenic testing, meaning that replacing uniform with risk-stratified screening would be cost-effective. However, colonoscopy uptake was assumed to remain constant in these analyses, which is uncertain. In sensitivity analyses we showed that if the increased complexity or potential problematic acceptability of a risk-stratified screening program leads to a reduced colonoscopy uptake, then benefits of risk stratification can easily be offset. Complexity and acceptability issues could partly be solved by including fewer risk categories and offering everyone at least one lifetime screen. However, sensitivity analyses showed that especially including fewer risk categories decreases the potential benefits of risk-stratified screening.

This study represents an early exploration of the potential benefit of risk-stratified screening based on common genetic variants. In the base-case analysis, we assumed full adherence both with genetic testing and subsequent recommended screening. In reality, people may refrain from polygenic testing for various well-grounded reasons (40), and if tested, may not be screened according to their optimal screening strategy. In a recent US survey among people with an intermediate familial CRC risk, three-fourths of participants said that they would probably (47%) or definitely (27%) have SNP testing to estimate their CRC risk (41). Actual uptake of polygenic testing in the general population will depend on the implementation of the program and the information provided to the public.

Although individuals with a family history of CRC are more adherent with CRC screening guidelines (42), increased awareness does not necessarily lead to increased adherence. One US study showed that knowledge of elevated CRC risk increased screening participation from 50% to 67% in whites, but, surprisingly, it decreased screening participation from 54% to 33% in individuals of other ethnicities (43). If implemented, screening based on polygenic risk should be organized with care, with adequate education and counseling before and after testing, so that potential decreases both in overall adherence and adherence within certain subgroups of the population could be minimized.

By varying the AUC value and other uncertain parameters, we considered a broad spectrum of potential current and future applications of risk-stratified screening. Nevertheless, this study has some limitations. First, we did not assume any disutility for having a polygenic test and for knowing your polygenic risk profile. Even though knowing that you have an increased CRC risk can be burdensome, it also enables people to get more intensive colonoscopy screening that could allow early detection of cancer. Moreover, the majority will be reassured by a relatively low CRC risk. Second, we presumed that our ability to predict risk for CRC is equally robust across the entire spectrum of risk (ie, for those at decreased risk or increased risk), which remains to be demonstrated empirically. Third, we did not assume any additional costs for risk-stratified screening compared with uniform screening, except for polygenic testing costs. Additional costs will likely include those of implementing the program (eg, planning and organizing both the introduction of polygenic testing and the switch to risk-stratified screening, educating health professionals and the general population), counseling eligible individuals before and after polygenic testing, and close monitoring of the program. Including those costs would have negatively affected the cost-effectiveness of risk-stratified screening, but because exact costs are currently unknown, quantifying the effect is not possible.

We showed that risk-stratified colonoscopy screening may become clinically relevant as more genetic variants associated with CRC risk are identified. Although AUC values above 0.75 may not seem feasible, it has been estimated that from the total number of SNPs associated with CRC risk, less than 10% have currently been identified (16). Another way of increasing the discriminatory ability of risk-stratification algorithms is by including other risk factors, such as sex and family history of CRC (18,44), and potentially also lifestyle (45). Efforts to develop multivariable risk models that include polygenic along with other established risk factors might offer more rapid progress toward risk-based screening for CRC rather than relying on polygenic risk models.

Other studies evaluating the merit of screening based on polygenic risk have considered a more simplified approach; for example, with individuals being invited for regular screening when their absolute disease risk was higher than a certain threshold (17,46). For the UK population, it was estimated that compared with uniform stool-based screening, risk-based screening would reduce the number of men and women being eligible for screening by 16% and 17%, respectively, at a cost of 10% and 8% fewer screen-detected CRC cases. Similar results have been found for breast and prostate cancer screening (46,47).

In conclusion, with the current discriminatory performance of polygenic testing, CRC screening based on polygenic risk is unlikely to be more cost-effective than uniform screening. This is expected to change with a greater than 0.05 increase in discriminatory test performance, a greater than 30% reduction in polygenic testing costs, or a greater than 5% increase in adherence with screening. Risk-stratification algorithms could further be enhanced by including other risk factors, such as lifestyle. In any case, risk-based screening would need to be implemented and monitored very carefully to ensure a continued colonoscopy uptake.

## Funding

This work was supported by the National Cancer Institute (grant number U01CA152959) as part of the Cancer Intervention and Surveillance Modeling Network (CISNET), with a supplement from the Evaluation of Genomic Applications in Practice and Prevention (EGAPP). Its contents are solely the responsibility of the authors and do not necessarily represent the official views of the National Cancer Institute or Centers for Disease Control and Prevention.

## Notes

Affiliations of authors: Department of Public Health, Erasmus MC, University Medical Center Rotterdam, Rotterdam, the Netherlands (SKN, MvB, ILV); Division of Cardiovascular Medicine, Vanderbilt University Medical Center, Nashville, TN (SK); Division of Health Policy & Management, University of Minnesota School of Public Health, Minneapolis, MN (KMK); Office of Public Health Genomics, Centers for Disease Control and Prevention, Atlanta, GA (WDD); Genomic Medicine Institute, Geisinger, Danville, PA (MSW); Memorial Sloan Kettering Cancer Center, New York, NY (AGZ); The Colorado Trust, Denver, CO (NC); Department of Science, Technology, and Society, Virginia Tech, Blacksburg, VA (DTZ); Department of Basic Science Education, Virginia Tech-Carilion School of Medicine, Roanoke, VA (DTZ); Department of Family Medicine and Public Health, University of California San Diego, La Jolla, CA (TGG); Center for Health Research, Kaiser Permanente, Portland, OR (EMW, KABG); Kaiser Permanente Washington Health Research Institute, Seattle, WA (NBH); Department of Clinical Genetics, EMGO Institute for Health and Care Research, Section Community Genetics, VU University Medical Center, Amsterdam, the Netherlands (ACJWJ); Department of Epidemiology, Rollins School of Public Health, Emory University, Atlanta, GA (ACJWJ).

Conflicts of interest: None to declare.

Acknowledgments: This work resulted from a collaboration between the Cancer Intervention and Surveillance Network (CISNET) and the EGAPP Working Group. The authors would like to thank Craig Parzynski, MS, of Yale University, who provided us with frequency data on hospitalizations for colonoscopy complications, and Luc Coffeng, MD, PhD, of Erasmus MC, who helped us link the underlying RR distribution to the RR distribution generated by a polygenic test (ie, using an elliptical copula in R).

## Supplementary Material

pkz086_Supplementary_Data
