# Supplementary material for: Cost-Effectiveness of Risk-Stratified Colorectal Cancer Screening Based on Polygenic Risk: Current Status and Future Potential
Source: JNCI Cancer Spectr. 2019 Oct 14;4(1):pkz086. doi: 10.1093/jncics/pkz086 (PMC6988584; doi:10.1093/jncics/pkz086)

Appendix Figure 1.

Distribution of polygenic relative risk that is assumed to be revealed by a polygenic test having an AUC value of 0.60 (i.e., current discriminatory performance) and of 0.65, …, 0.80 (i.e., potential future discriminatory performance).

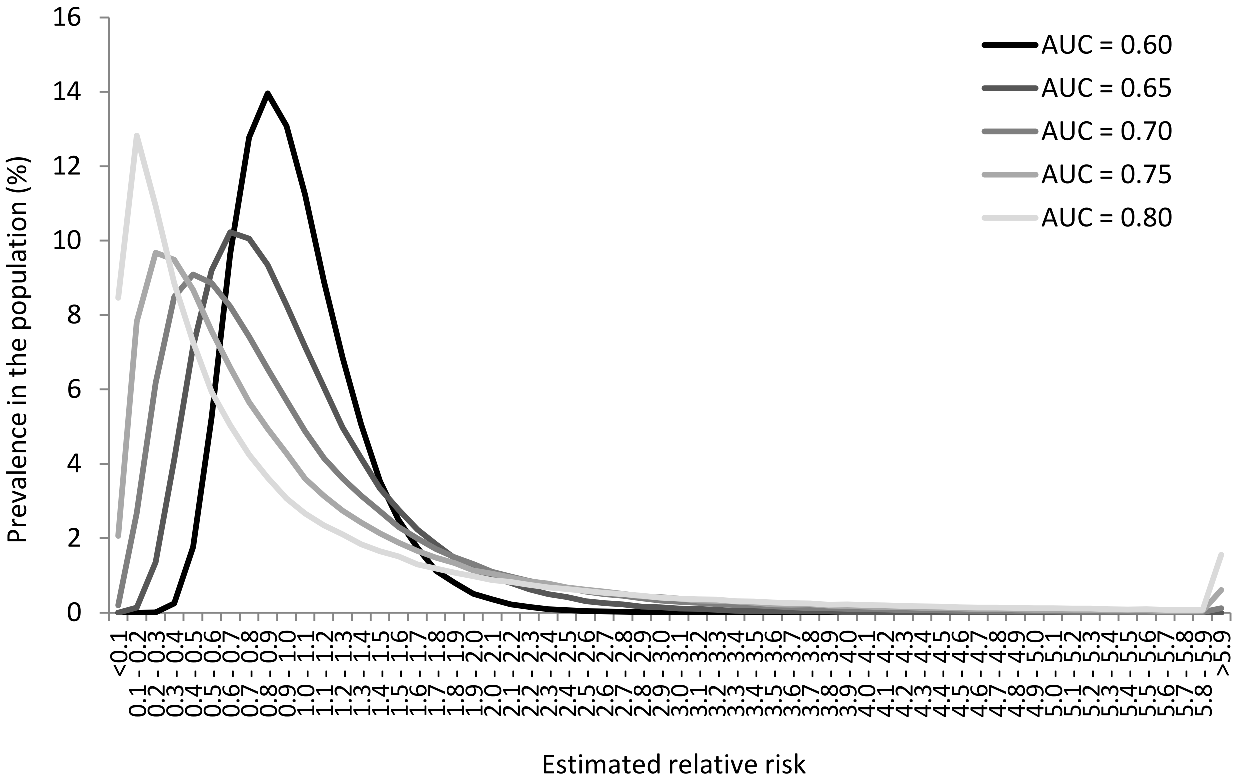

AUC = area under the receiver operating characteristic curve.

Appendix Figure 2.

Risk-stratified screening strategies by relative risk as estimated by a polygenic test with AUC value of 0.60, 0.65, 0.70, 0.75 and 0.80, given a willingness-to-pay threshold for risk-stratified screening that ensures that the entire risk-stratified screening program yields as least as many QALYs as a uniform screening program with colonoscopies at ages 50, 60 and 70.* Relative risk groups for which a no screening strategy was optimal in the base-case analysis were given the strategy that was cost effective after eliminating the no screening strategy. For every strategy, the number of lifetime colonoscopies, screening interval and age range of screening is given (i.e. “3 COLs, every 10y, ages 50-75” refers to 3 lifetime colonoscopies with an interval of 10 years in individuals aged 50-75).

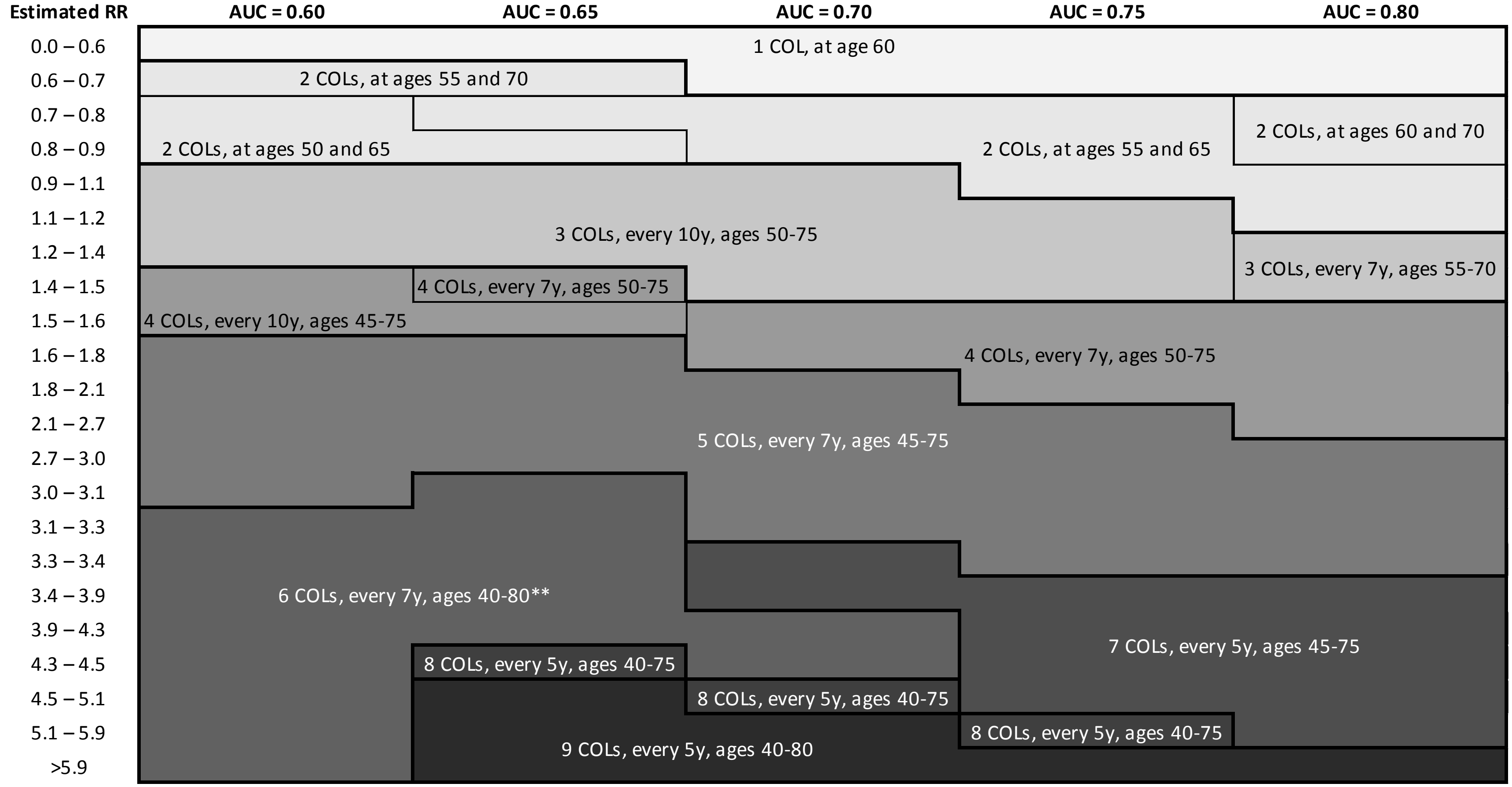

RR = relative risk; AUC = area under the receiver operating characteristic curve; COLs = colonoscopies; y = years.

* Willingness-to-pay threshold equals $69,000, $65,000, $52,800, $41,000, and $31,000 for AUC = 0.60, 0.65, 0.70, 0.75, and 0.80, respectively.

** For AUC = 0.70, individuals with an estimated RR of 3.9-4.5 are offered fewer lifetime screens than those with an estimated RR of 3.3-3.9, but the age range in which they are offered screening is broader.

Appendix Figure 3.

Risk-stratified screening strategies by relative risk as estimated by a polygenic test with AUC value of 0.60, 0.65, 0.70, 0.75 and 0.80, given a willingness-to-pay threshold for risk-stratified screening that ensures that the entire risk-stratified screening program yields as least as many QALYs as a uniform screening program with colonoscopies at ages 50, 60 and 70.* Relative risk groups were merged into three risk groups, that were as equal in size as possible. For every strategy, the number of lifetime colonoscopies, screening interval and age range of screening is given (i.e. “3 COLs, every 10y, ages 50-75” refers to 3 lifetime colonoscopies with an interval of 10 years in individuals aged 50-75).

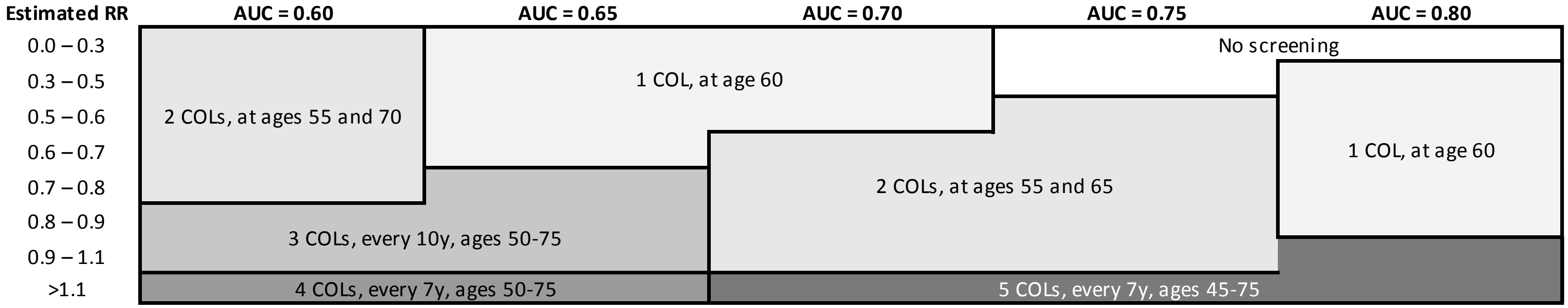

RR = relative risk; AUC = area under the receiver operating characteristic curve; COLs = colonoscopies; y = years.

* Willingness-to-pay threshold equals $73,000, $63,000, $51,700, $43,000, and $40,000 for AUC = 0.60, 0.65, 0.70, 0.75, and 0.80, respectively.

Appendix Table 1.

Alternative costs of colorectal cancer screening and treatment, based on commercial rates rather than Medicare reimbursement.

COSTS (2014 US$)[a,b]				
**Per colonoscopy**				
without polypectomy/biopsy	1,681			
with polypectomy/biopsy	2,055			
**Per complication of colonoscopy**				
Serious gastrointestinal event[c]	13,893			
Other gastrointestinal event[d]	9,668			
Cardiovascular event[e]	10,693			
**Per LY with CRC care**[f]	Initial care	Continuing care	Terminal care *Death CRC*	Terminal care *Death other cause*
Stage I CRC	48,336	4,077	84,857	24,406
Stage II CRC	65,334	3,822	84,514	21,837
Stage III CRC	79,588	5,374	89,235	27,495
Stage IV CRC	104,010	16,453	118,120	65,973

QALY = quality-adjusted life year; LY = life year; CRC = colorectal cancer.

a Costs include copayments and patient time costs (i.e. the opportunity costs of spending time on screening or being treated for a complication or CRC), but do not include travel costs, costs of lost productivity, and unrelated health care and non-health care costs in added years of life. We assumed that the value of patient time was equal to the median wage rate in 2014: $17.09 per hour.[46] We assumed that colonoscopies used up 36 hours, serious gastrointestinal complications 192 hours, other gastrointestinal complications 96 hours and cardiovascular complications 120 hours of patient time. Patient time costs associated with CRC care were provided by Yabroff (personal communication), and were calculated using the methodology described in a study by Yabroff and colleagues.[47]

b Commercial rates were determined by multiplying healthcare related cost components (i.e., copayments and Medicare reimbursement) with a factor of 1.35 as recently suggested by Ladabaum et al.[33] Patient time costs were not considered to differ between commercial rates and Medicare reimbursement.

c Serious gastrointestinal events are perforations, gastrointestinal bleeding, or transfusions.

d Other gastrointestinal events are paralytic ileus, nausea and vomiting, dehydration, or abdominal pain.

e Cardiovascular events are myocardial infarction or angina, arrhythmias, congestive heart failure, cardiac or respiratory arrest, syncope, hypotension, or shock.

f Care for CRC was divided in three clinically relevant phases: the initial, continuing, and terminal care phase. The initial care phase was defined as the first 12 months after diagnosis; the terminal care phase was defined as the final 12 months of life; the continuing care phase was defined as all months in between. In the terminal care phase, we distinguished between CRC patients dying from CRC and CRC patients dying from another cause. For patients surviving less than 24 months, the final 12 months were allocated to the terminal care phase and the remaining months were allocated to the initial care phase.

Appendix Table 2.

Effects and costs, based on commercial rates (Appendix Table 1), per 1,000 40-year-old individuals for no screening, uniform screening with colonoscopy at ages 50, 60 and 70, and risk-stratified screening, given a willingness-to-pay threshold for risk-stratified screening that ensures that the entire risk-stratified screening program yields as least as many QALYs as a uniform screening program with colonoscopies at ages 50, 60 and 70.[a]

	Colonoscopies[b]		CRC cases		CRC deaths		Life years[c]		QALYs[c]		Costs, USD (*1,000)[c,d]							
											Polygenic testing		CRC screening[e]		Cancer diagnosis and treatment		Total	
**No screening**	67		67		28		22,940		22,907		0		5		3,259		3,265	
**Uniform screening[f]**	3,247	(ref)	30	(ref)	8	(ref)	23,014	(ref)	22,993	(ref)	0	(ref)	3,352	(ref)	1,705	(ref)	5,057	(ref)
**Risk-stratified screening[g]**	3,116	(-131)	28	(-1)	8	(-1)	23,013	(-0)	22,993	(+0)	200	(+200)	3,197	(-155)	1,692	(-13)	5,089	(+32)

AUC = area under the receiver operating characteristic curve; CRC = colorectal cancer; QALYs = quality-adjusted life years; ref = reference value; (n) = increase/decrease compared to uniform screening.

a Willingness-to-pay threshold equals $79,800 per QALY.

b Includes screening colonoscopies, surveillance colonoscopies, and diagnostic colonoscopies.

c (Quality-adjusted) life years and costs were discounted at an annual rate of 3%.

d Costs are in 2014 U.S. Dollars.

e Includes costs of screening colonoscopies, surveillance colonoscopies, and colonoscopy complications.

f Uniform screening was defined as colonoscopy screening at ages 50, 60 and 70 for all.

g Risk-stratified screening with a base case AUC value of polygenic testing of 0.60, and base case costs of polygenic testing of $200 per person.

MODEL APPENDIX – MISCAN-Colon

General Model Structure

MISCAN-Colon is a stochastic microsimulation model for colorectal cancer (CRC) programmed in Delphi (Borland Software Corporation, Scotts Valley, California, United States). It can be used to explain and predict trends in CRC incidence and mortality and to quantify the effects and costs of primary prevention of CRC, screening for CRC, and surveillance after polypectomy.

The term 'microsimulation' implies that individuals are moved through the model one at a time, rather than as proportions of a cohort. This allows future state transitions to depend on past transitions, giving the model a 'memory'. Furthermore, unlike most traditional Markov models, MISCAN-Colon does not use yearly transition probabilities; instead it generates durations in states, thereby increasing model flexibility and computational performance. The term 'stochastic' implies that the model simulates sequences of events by drawing from distributions of probabilities/durations, rather than using fixed values. Hence, the results of the model are subject to random variation.

MISCAN-Colon consists of 3 modules: a demography module, natural history module, and screening module.

The Demography Module

Using birth-tables and life-tables representative for the population under consideration, MISCAN-Colon draws a date of birth and a date of non-CRC death for each individual simulated. In MISCAN-Colon the maximum age an individual can achieve is exactly 100 years.

The Natural History Module

Transitions

As each simulated person ages, one or more adenomas may develop (**Model Appendix Figure 1**). These adenomas can be either progressive or non-progressive. Both progressive and non-progressive adenomas can grow in size from small (≤5mm), to medium (6-9mm), to large (≥10mm); however, only progressive adenomas can develop into preclinical cancer. A preclinical cancer may progress through stages I to IV; however, during each stage CRC may be diagnosed because of symptoms. After clinical diagnosis, CRC survival is simulated using age-, stage-, and localization-specific survival estimates for clinically diagnosed CRC obtained from a study by Rutter and colleagues.[1] For individuals with synchronous CRCs at time of diagnosis, the survival of the most advanced cancer is used. The date of death for individuals with CRC is set to the earliest simulated death (either due to CRC or due to another cause (see: 'The demography module')).

Transition Probabilities and Durations in States

An individual's risk of developing adenomas depends on the individual's age and a personal risk index. As a result of the latter most individuals develop no adenomas, whilst some develop many. We assumed that the distribution of adenomas over the colon and rectum equals the distribution of cancers as observed in SEER before the introduction of screening.[2] The age-specific onset of adenomas and the dispersion of the personal risk index were calibrated to data on the prevalence and multiplicity distribution of adenomas as observed in autopsy studies (**Model Appendix Figure 2**).[3-12] The age-specific probability of adenoma-progressivity and the age- and localization-specific transition probabilities between preclinical cancer stages and between preclinical and clinical cancer stages were simultaneously calibrated to SEER data on the age-, stage-, and localization-specific incidence of CRC as observed before the introduction of screening (**Model Appendix Figure 3**).[2]

The model incorporates a large variability in progression rates, enabling some adenomas to progress to CRC within a few years. The average durations of the preclinical cancer stages were calibrated to the rates of screen-detected and interval cancers observed in randomized controlled trials evaluating screening using guaiac fecal occult blood tests.[13-15] This exercise has been described extensively in a publication by Lansdorp-Vogelaar and colleagues.[16] The average duration from the emergence of an adenoma (state 2) until progression into preclinical cancer (state 7) (i.e. the adenoma dwell-time) was calibrated to the rates of interval cancers (including surveillance detected cancers) observed in a randomized controlled trial evaluating once-only sigmoidoscopy screening (**Model Appendix Figure 4**).[17] We assumed an equal overall dwell-time for adenomas developing into CRC from a medium size (30% of all CRCs) and from a large size (70% of all CRCs). All durations in the adenoma and preclinical cancer phase were drawn from exponential distributions. Durations within the adenoma phase and within the preclinical cancer phase were assumed to be perfectly correlated (i.e. if a small adenoma grows into a medium-sized adenoma rapidly, it will also grow into a large adenoma or develop into CRC rapidly); however, durations in the adenoma phase were assumed to be uncorrelated with durations in the preclinical cancer phase (i.e. a rapidly growing adenoma does not necessarily develop into a rapidly progressing cancer). The proportion of medium sized, non-progressive adenomas growing large and the average duration in the medium size, non-progressive adenoma state (state 5) were calibrated to size-specific adenoma detection rates observed in a Dutch randomized controlled trial on colonoscopy screening (*data not shown*).

The Screening Module

Screening will alter some of the simulated life histories: Some cancers will be prevented by the detection and removal of adenomas; other cancers will be detected in an earlier stage with a more favorable survival. As the stage-specific survival of screen-detected CRC as observed in randomized

controlled trials on guaiac fecal occult blood testing was substantially more favorable than that of clinically detected CRC, even after correcting for lead-time bias,[16] we assigned those screen-detected cancers that would have been clinically detected in the same stage the survival corresponding to a one stage less progressive cancer. Hence, a cancer screen-detected in stage II, that would also have been clinically diagnosed in stage II, is assigned the survival of a clinically diagnosed stage I cancer. The only exceptions were screen-detected stage IV cancers. These cancers were always assigned the survival of a clinically diagnosed stage IV cancer.

Besides modeling positive health effects of screening, we also model colonoscopy-related complications and over-diagnosis and over-treatment of CRC (i.e. the detection and treatment of cancers that would not have been diagnosed without screening).[18-20]

Integrating Modules

The demography module generates a date of birth and a date of non-CRC death for each individual simulated, creating a life-history without adenomas or CRC. In Patient A in **Model Appendix Figure 5**, the natural history module generates an adenoma. This adenoma progresses into preclinical cancer, which is diagnosed because of symptoms in stage II and results in CRC death before non-CRC death would have occurred. In the screening module a screening examination is simulated, indicated by the blue arrow. During this examination the adenoma is detected, and as a result both CRC and CRC death are prevented. Hence, in Patient A, screening prolongs life by the amount indicated by the green arrow. Patient B also develops an adenoma, and although this adenoma does progress into preclinical cancer, Patient B would never have been diagnosed with CRC in a scenario without screening (see *life history 2*). However, during the screening examination simulated in the screening module, again indicated by the blue arrow, CRC is screen-detected in stage I. Hence, in this patient screening results in over-diagnosis of CRC: It detects a cancer that would never have been diagnosed in a scenario without

screening. Hence, screening does not prolong life, but it does result in additional LYs with CRC care (over-treatment) as indicated by the red arrow.

References

1. Rutter CM, Johnson EA, Feuer EJ, Knudsen AB, Kuntz KM, Schrag D. Secular trends in colon and rectal cancer relative survival. *J Natl Cancer Inst* 2013; **105**(23): 1806-13.
2. SEER*Stat Database: Incidence–SEER 9 Regs Limited-Use NS-. Surveillance, Epidemiology, and End Results (SEER) Program. 2003. http://www.seer.cancer.gov (accessed July 1st, 2016.
3. Blatt LJ. Polyps of the colon and rectum: Incidence and distribution. *Dis Colon Rectum* 1961; **4**(4): 277-82.
4. Arminski TC, McLean DW. Incidence and Distribution of Adenomatous Polyps of the Colon and Rectum Based on 1,000 Autopsy Examinations. *Dis Colon Rectum* 1964; **7**: 249-61.
5. Bombi JA. Polyps of the colon in Barcelona, Spain. An autopsy study. *Cancer* 1988; **61**(7): 1472-6.
6. Chapman I. Adenomatous polypi of large intestine: incidence and distribution. *Ann Surg* 1963; **157**: 223-6.
7. Clark JC, Collan Y, Eide TJ, et al. Prevalence of polyps in an autopsy series from areas with varying incidence of large-bowel cancer. *Int J Cancer* 1985; **36**(2): 179-86.
8. Jass JR, Young PJ, Robinson EM. Predictors of presence, multiplicity, size and dysplasia of colorectal adenomas. A necropsy study in New Zealand. *Gut* 1992; **33**(11): 1508-14.
9. Johannsen LG, Momsen O, Jacobsen NO. Polyps of the large intestine in Aarhus, Denmark. An autopsy study. *Scand J Gastroenterol* 1989; **24**(7): 799-806.
10. Rickert RR, Auerbach O, Garfinkel L, Hammond EC, Frasca JM. Adenomatous lesions of the large bowel: an autopsy survey. *Cancer* 1979; **43**(5): 1847-57.
11. Vatn MH, Stalsberg H. The prevalence of polyps of the large intestine in Oslo: an autopsy study. *Cancer* 1982; **49**(4): 819-25.
12. Williams AR, Balasooriya BA, Day DW. Polyps and cancer of the large bowel: a necropsy study in Liverpool. *Gut* 1982; **23**(10): 835-42.
13. Hardcastle JD, Chamberlain JO, Robinson MH, et al. Randomised controlled trial of faecal-occult-blood screening for colorectal cancer. *Lancet* 1996; **348**(9040): 1472-7.
14. Jorgensen OD, Kronborg O, Fenger C. A randomised study of screening for colorectal cancer using faecal occult blood testing: results after 13 years and seven biennial screening rounds. *Gut* 2002; **50**(1): 29-32.
15. Mandel JS, Church TR, Ederer F, Bond JH. Colorectal cancer mortality: effectiveness of biennial screening for fecal occult blood. *J Natl Cancer Inst* 1999; **91**(5): 434-7.
16. Lansdorp-Vogelaar I, van Ballegooijen M, Boer R, Zauber A, Habbema JD. A novel hypothesis on the sensitivity of the fecal occult blood test: Results of a joint analysis of 3 randomized controlled trials. *Cancer* 2009; **115**(11): 2410-9.
17. Atkin WS, Edwards R, Kralj-Hans I, et al. Once-only flexible sigmoidoscopy screening in prevention of colorectal cancer: a multicentre randomised controlled trial. *Lancet* 2010; **375**(9726): 1624-33.
18. Warren JL, Klabunde CN, Mariotto AB, et al. Adverse events after outpatient colonoscopy in the Medicare population. *Ann Intern Med* 2009; **150**(12): 849-57, W152.
19. Gatto NM, Frucht H, Sundararajan V, Jacobson JS, Grann VR, Neugut AI. Risk of perforation after colonoscopy and sigmoidoscopy: a population-based study. *J Natl Cancer Inst* 2003; **95**(3): 230-6.
20. van Hees F, Zauber AG, Klabunde CN, Goede SL, Lansdorp-Vogelaar I, van Ballegooijen M. The Appropriateness of More Intensive Colonoscopy Screening Than Recommended in Medicare Beneficiaries - A Modeling Study. *JAMA Intern Med* 2014; **174**(10): 1568-76.

Model Appendix Figure 1. **An Overview of the Natural History Module of MISCAN-Colon.**

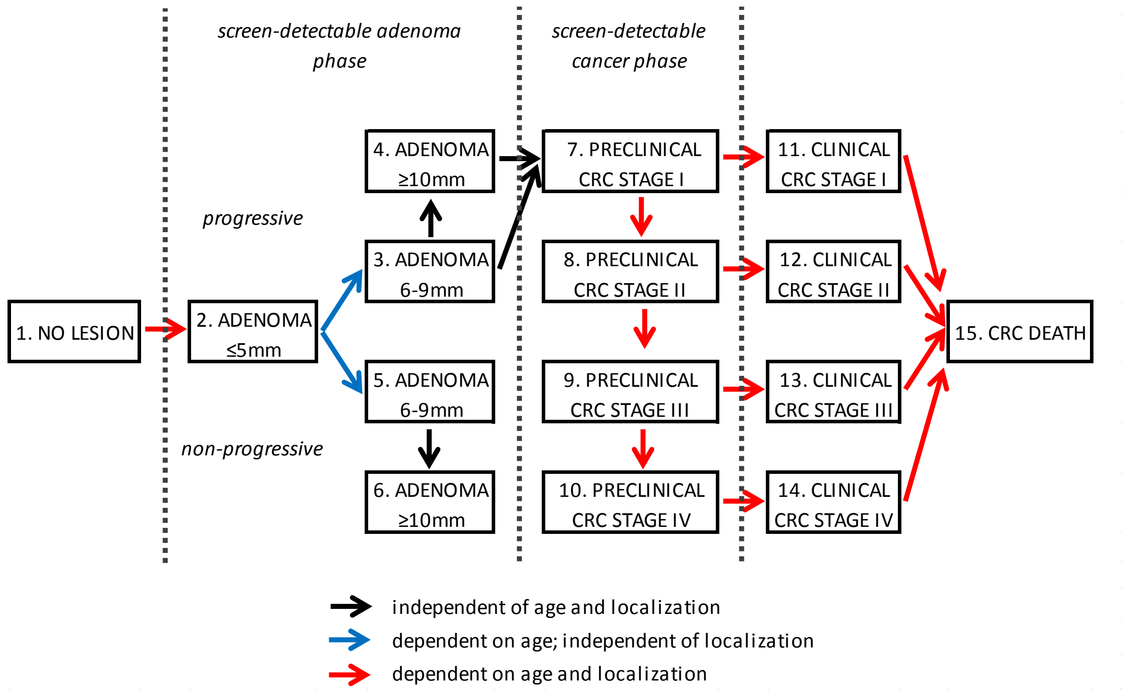

Model Appendix Figure 2. **Adenoma Prevalence Observed in Selected Autopsy Studies Versus Simulated by MISCAN-Colon (% of individuals with adenomas).***

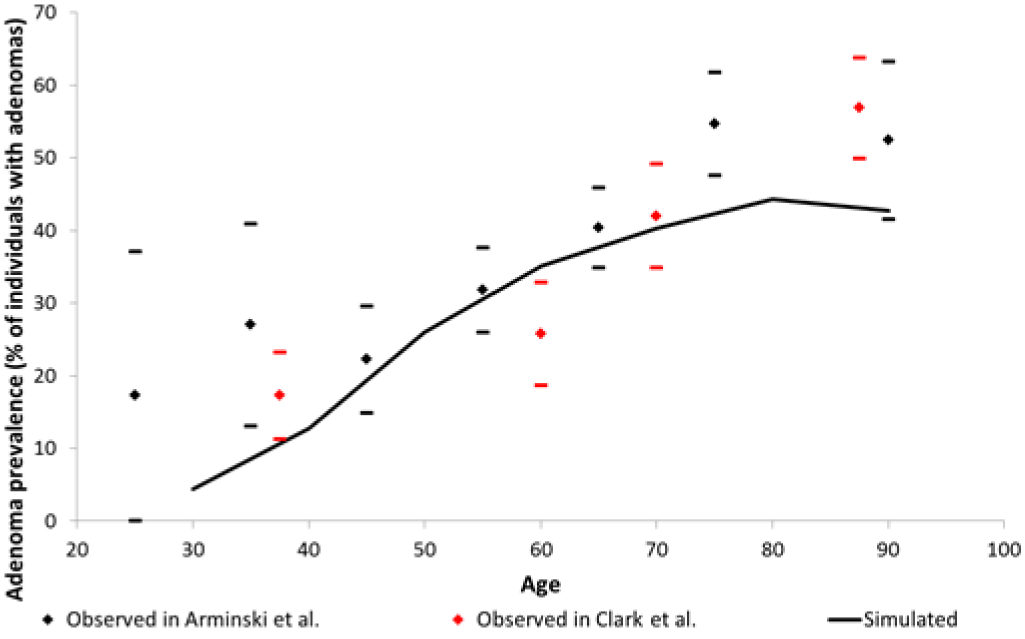

*Observed results are only shown for the two largest studies (Arminski et al.[4] and Clark et al.[7]) on which the model has been calibrated. MISCAN-Colon has additionally been calibrated to 8 other autopsy studies.[3,5,6,8-12]

Model Appendix Figure 3. **CRC Incidence Observed Before the Introduction of Screening Versus Simulated by MISCAN-Colon (total (A), stage I CRC (B), stage II CRC (C), stage III CRC (D), stage IV CRC (E); cases per 100,000 person years).**

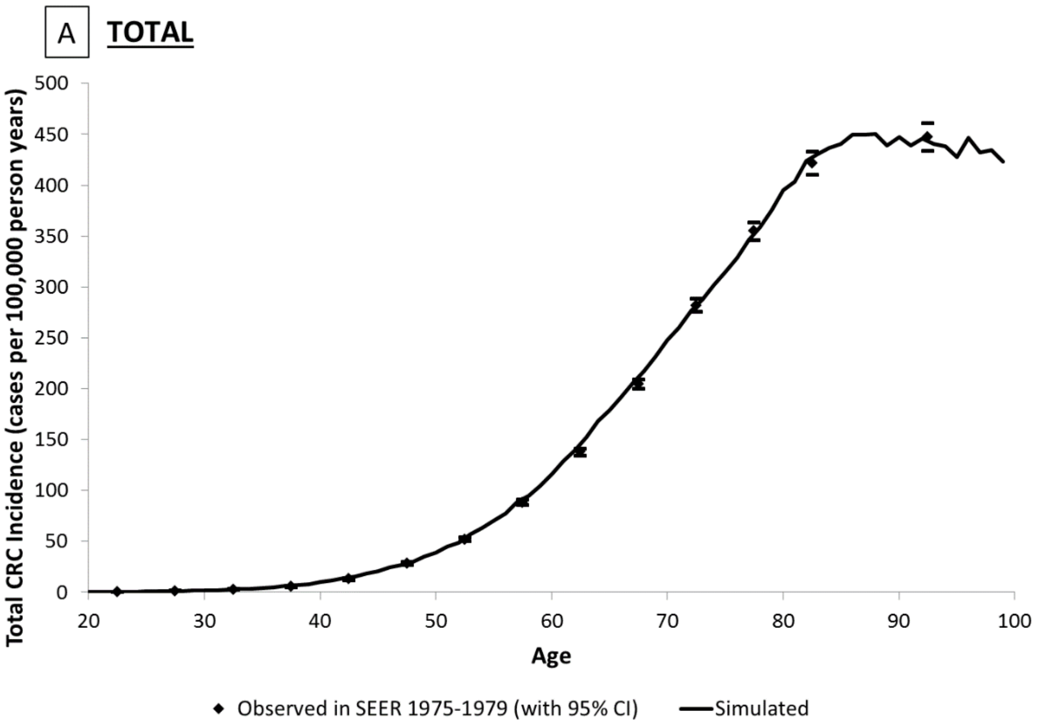

B **STAGE I CRC**

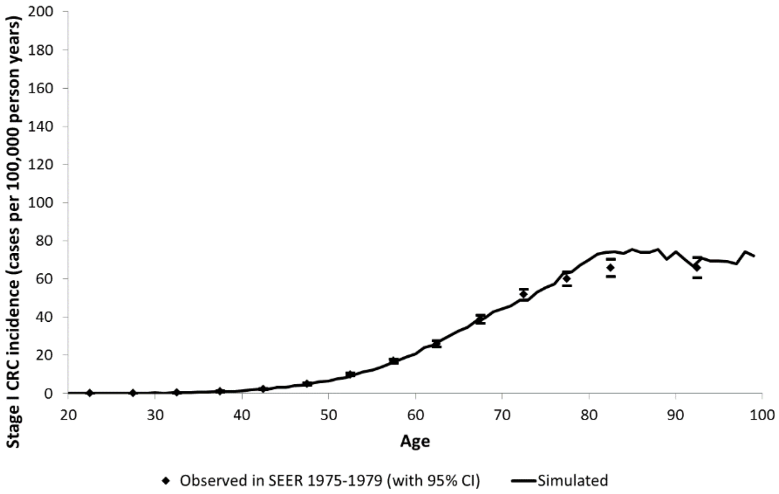

C **STAGE II CRC**

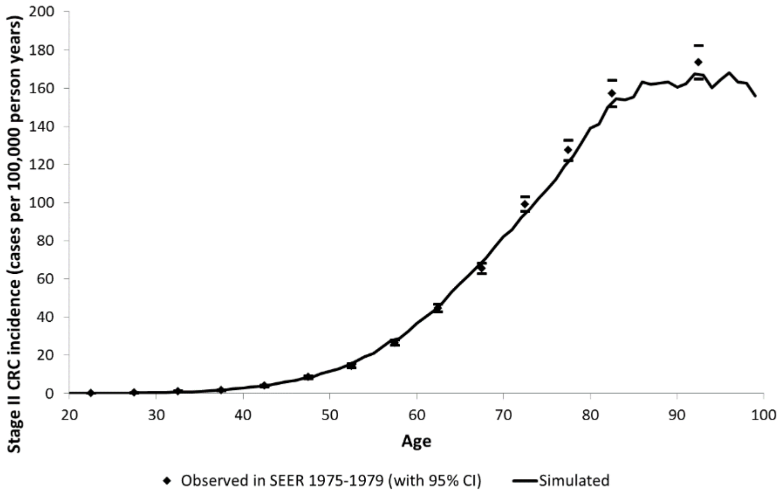

D **STAGE III CRC**

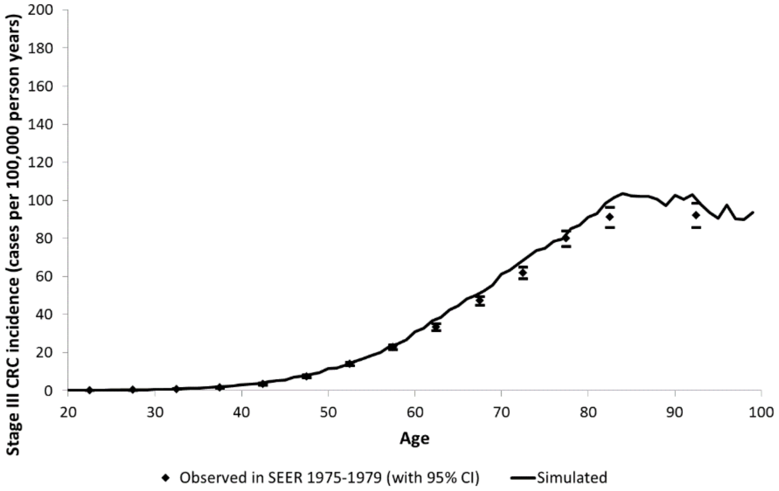

E **STAGE IV CRC**

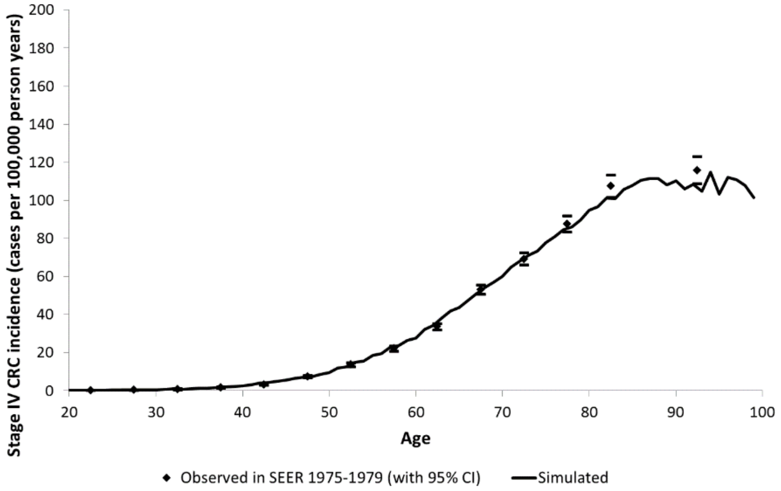

Model Appendix Figure 4. **Distal CRC Incidence Observed in the Intervention Group of the UK Flexible Sigmoidoscopy Trial Versus Simulated by MISCAN-Colon (per year of follow-up (A), cumulative (B); cases per 100,000 person years).**

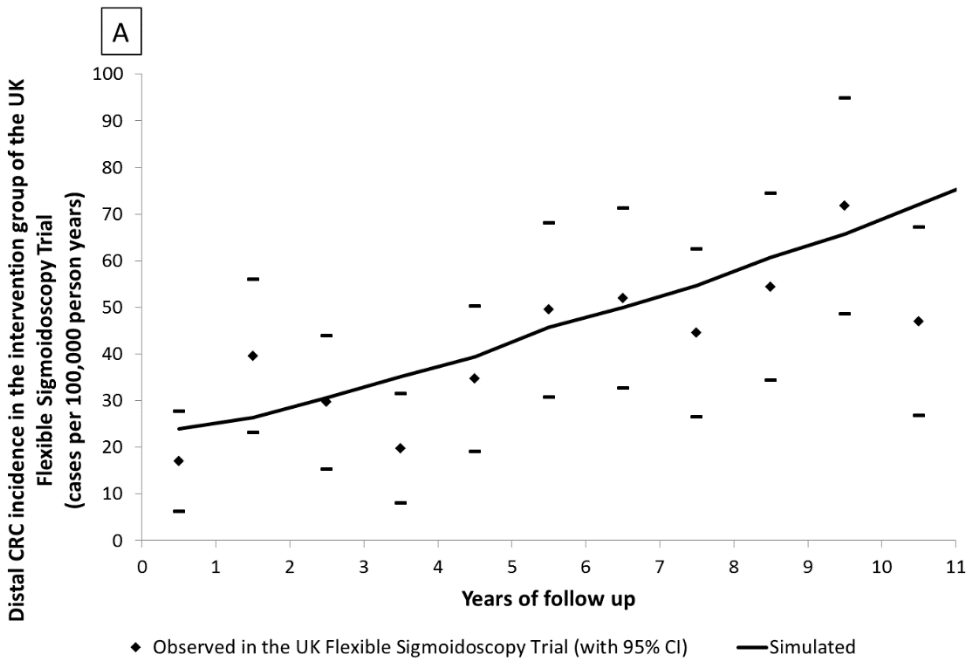

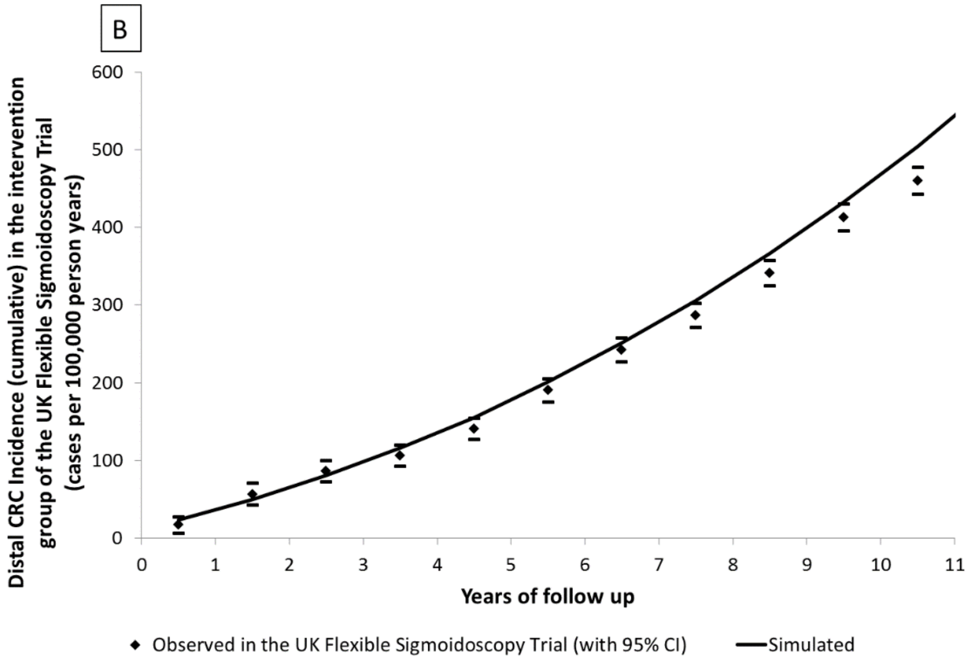

Model Appendix Figure 5. **Integrating Modules: Two example Patients.**

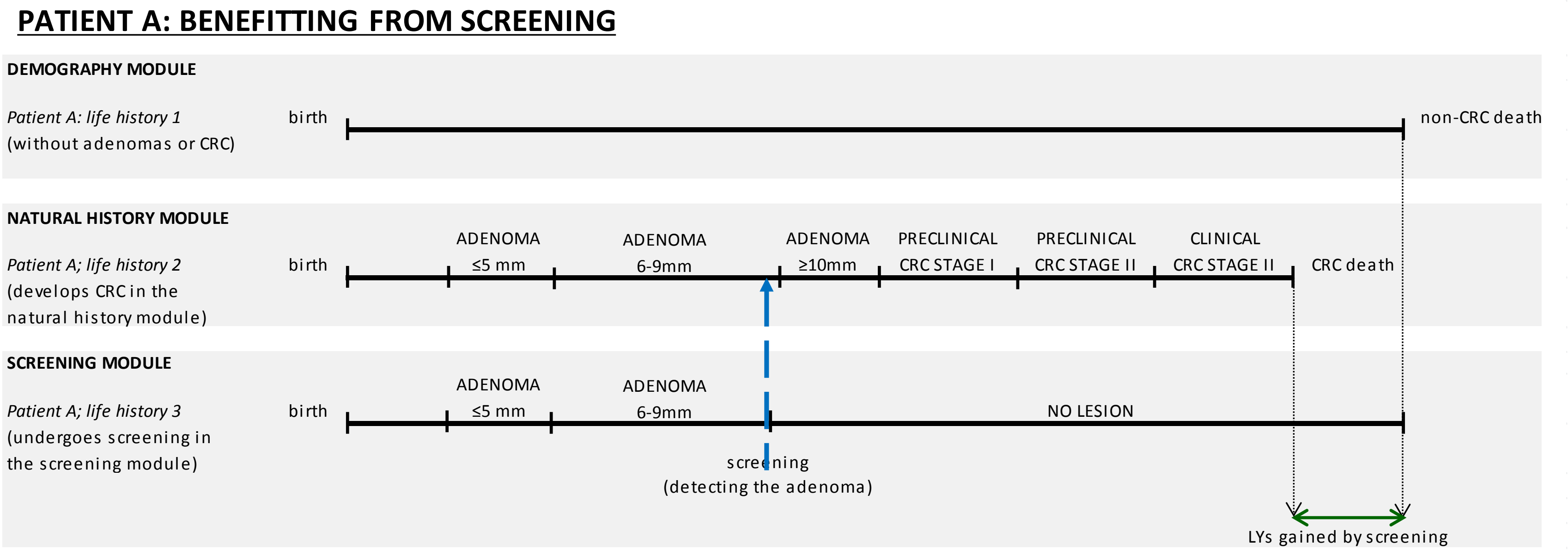

PATIENT B: OVER-DIAGNOSING CRC

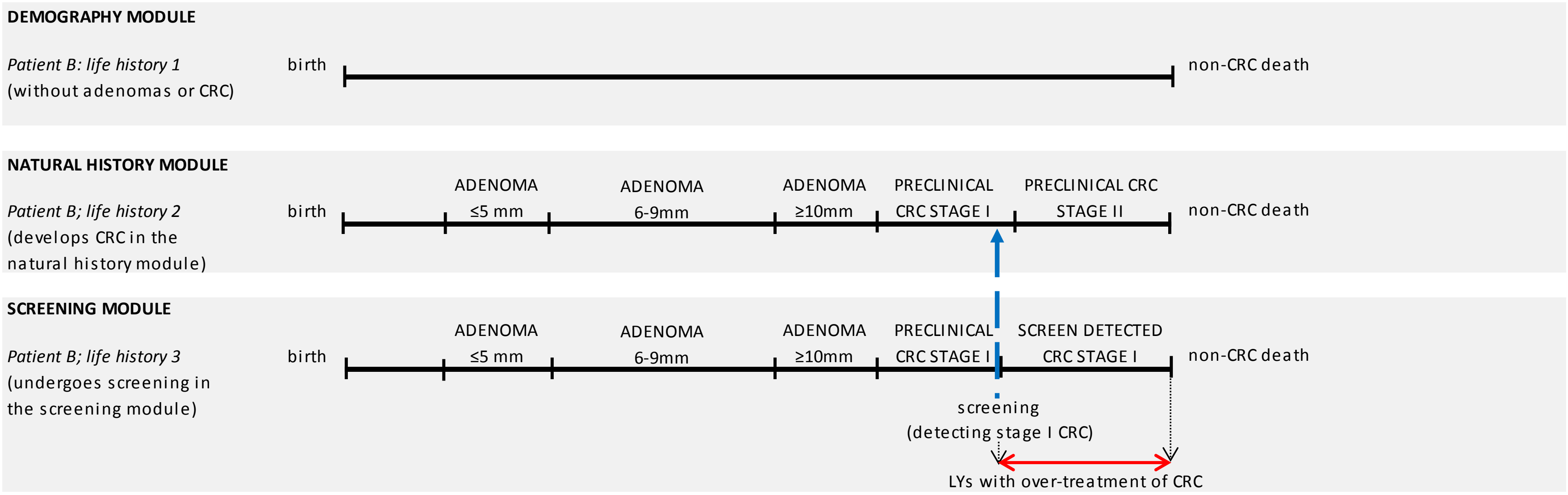

TECHNICAL APPENDIX – Modeling Colorectal Cancer Screening based on Polygenic Risk in the MISCAN-Colon Model

This file describes the approach (1) to model the increased risk of colorectal cancer (CRC) as conferred through polygenic risk factors and (2) to estimate the cost-effectiveness of CRC screening based on polygenic risk factors compared to uniform CRC screening. The approach consists of five consecutive steps.

Step 1. Modeling underlying Baseline Polygenic Risk

In the Microsimulation Screening Analysis (MISCAN)-Colon model, each simulated individual is given a lifetime underlying baseline risk for CRC at birth. This risk may be regarded as someone's underlying genetic predisposition for developing CRC. When we simulate a population excluding individuals with known single genetic mutations associated with CRC risk, the lifetime risk reflects the polygenic risk, i.e. the risk of developing CRC based on common genetic variants. Whether a simulated person actually develops CRC is based on both this underlying polygenic risk and on a random component. The random component represents not only the unmodifiable 'bad luck' factor that is associated with the formation of cancer cells, but also variable exposure to lifestyle and environmental risk factors. The polygenic risk is assumed to come from a Gamma distribution with mean 1 and variance 1.98628, based on model calibration (see Model Appendix for details). For this analysis, we split the simulated population into 60 different relative risk groups based on their relative polygenic risk compared to the average population, with relative risks varying from 0–0.1, 0.1–0.2, …, 5.7–5.8, and 5.8–5.9 to >5.9 (Figure 1).

Step 2. Estimating Polygenic Relative Risk using polygenic testing

The underlying polygenic risk in the population is generally not known, but a polygenic test may be used to estimate this. The discriminatory performance of the polygenic test to distinguish low-risk from high-risk individuals, is expressed by the area under the receiver operating characteristic (ROC) curve (i.e., AUC), and determines the usefulness of such a test. For example, if all individuals would have an estimated relative risk of 1, the polygenic test would not be able to discriminate between low-risk and high-risk individuals at all. In this case, the AUC would equal its minimum value of 0.50. Alternatively, if a polygenic test would include all single nucleotide polymorphisms (SNPs) that are associated with CRC risk (including SNPs that have not been identified), then the polygenic test would be able to reveal the underlying polygenic risk of all simulated individuals. This does not mean that it would be able to tell who will certainly develop CRC in the future and who will not. This depends on all risk factors for developing CRC (lifestyle, environment, “bad luck”), and not just SNPs. So, even if all possible SNPs would be included in the polygenic test, its AUC would not equal 1. In MISCAN-Colon, the maximum AUC would be ~0.8325, based on assumptions for the underlying polygenic risk distribution and the random risk component in MISCAN-Colon.

Currently, a fraction of all SNPs associated with CRC risk have been identified. The AUC value of polygenic testing based on these SNPs is approximately 0.6.

Previous research has shown that if an AUC for a predictive test has been obtained from a multitude of factors that each have a very modest contribution to the AUC, the distribution of estimated relative risk over the population always follows the same pattern (Janssens, Genet. Med 2006). Using the methodology derived in this paper, we generated a distribution of estimated relative risk of CRC over the population. In short, we constructed a hypothetical population with a predicted risk for each individual using an iterative procedure in which we added as many genetic variants until the AUC

reached a pre-specified value. We then divided the predicted risk of each individual by the population disease prevalence to transform to the corresponding relative risk, which was finally categorized into 60 groups.

As expected, the higher the AUC value, the smaller the number of individuals with an estimated polygenic relative risk (RR) close to 1, and the larger the number of individuals with either a relatively high polygenic RR (i.e., likely to develop CRC) or a relatively low polygenic RR (i.e., not likely to develop CRC). Figure 2 shows the estimated polygenic RR distribution for polygenic tests having an AUC value of 0.60, 0.65, 0.70, 0.75 and 0.80, generated this way.

Step 3. Modeling the concordance between estimated and underlying Polygenic Relative Risk

In Steps 1 and 2 we have identified the distribution of the underlying polygenic risk in the population and the estimated polygenic risk distribution, respectively, i.e., we have two marginal distributions in the same population. In Step 3, we use an elliptical Copula approach to generate a joint distribution of the underlying and estimated polygenic risk ensuring that both marginal distributions are maintained. For each AUC value, the Copula assigns a distribution of *underlying* polygenic risk values to each of the 60 estimated polygenic risk categories. As an example, Figure 3 shows the underlying polygenic risk distribution for individuals with an estimated polygenic RR between 0.7 and 0.8, when using a polygenic test with AUC = 0.60. Taken together, these underlying polygenic risk distributions add up to the overall distribution of polygenic risk as shown in Figure 1.

A graphical representation of the elliptical copula is presented in Figure 4. This figure shows the concordance between the underlying polygenic RR and the polygenic RR as estimated by the polygenic test for 10,000 simulated individuals. For the analysis, we determined the concordance of the two distributions for 10 million simulated individuals, but plotting this many symbols in one figure did not visualize the elliptical copula very well.

The parameters for the Copula were chosen such that, for each estimated polygenic risk group, the average of the underlying polygenic risks of individuals was within the bounds of the estimated polygenic risk. For the example in Figure 3, with estimated polygenic risk between 0.7 and 0.8, the average underlying polygenic risk was equal to 0.722.

Step 4. Estimating the Benefits and Costs of Different Screening Strategies

For every underlying polygenic risk cohort, consisting of >1,000,000 simulated individuals, we evaluate 126 different colonoscopy screening strategies, varying in:

- The start age of screening: 40, 45, 50, 55, 60, and 65 years
- The end age of screening: 70, 75, 80, and 85 years
- The screening interval: 1, 2, 3, 5, 7, 10, 15, and 20 years

We calculate the costs and quality-adjusted life years (QALYs) gained of each of these strategies as compared to the situation in which the underlying polygenic risk cohort would not be screened.

We then use those costs and QALYs of all underlying polygenic risk cohorts to calculate the costs and QALYs for each estimated polygenic risk group. This is done by taking a weighted average of the results for the underlying polygenic risk cohorts, using their distribution for the estimated polygenic risk group as described in Step 3. For example, for those with an estimated polygenic risk between 0.7 and 0.8 (see Figure 3), the costs and QALYs of a particular screening strategy are calculated by taking 31.3%, 11.1%, and 7.8% of the costs and QALYs of offering this screening strategy to those with an underlying polygenic RR between 0.0–0.1, 0.1–0.2, and 0.2–0.3, respectively. The costs and QALYs of those with an underlying polygenic RR from 0.7–0.8 only account for 3.0% of the total costs and QALYs.

Step 5. Optimal risk-stratified screening based on polygenic test results

For each estimated polygenic risk group, we then identify the efficient screening strategies, that is, strategies that do not have an alternative or combination of alternatives that result in more QALYs at the same or lower costs. This results in a set of efficient strategies for each risk group. For every efficient strategy, we determine the incremental cost-effectiveness ratio (ICER), which is calculated as the incremental costs per incremental life-year gained compared with the next less cost-efficient strategy. For all risk groups, the strategy with an ICER value closest to but not exceeding a certain willingness-to-pay threshold (e.g. $100,000 per QALY gained) is considered the optimal strategy. Costs and QALY gained are discounted at 3% per year.

After we have identified the optimal colonoscopy screening strategy for each individual risk group, we sum the individual estimates of all risk groups to obtain the population-level costs and benefits. We can use these population estimates to compare costs and benefits of offering the total population the same colonoscopy screening strategy (as currently recommended) versus risk-stratified colonoscopy screening based on polygenic risk. This comparison informs us about whether risk-stratified screening based on polygenic risk is worthwhile from a cost-effectiveness perspective.

Technical Appendix Figure 1. **Subdivision of simulated population in polygenic relative risk cohorts in MISCAN.**

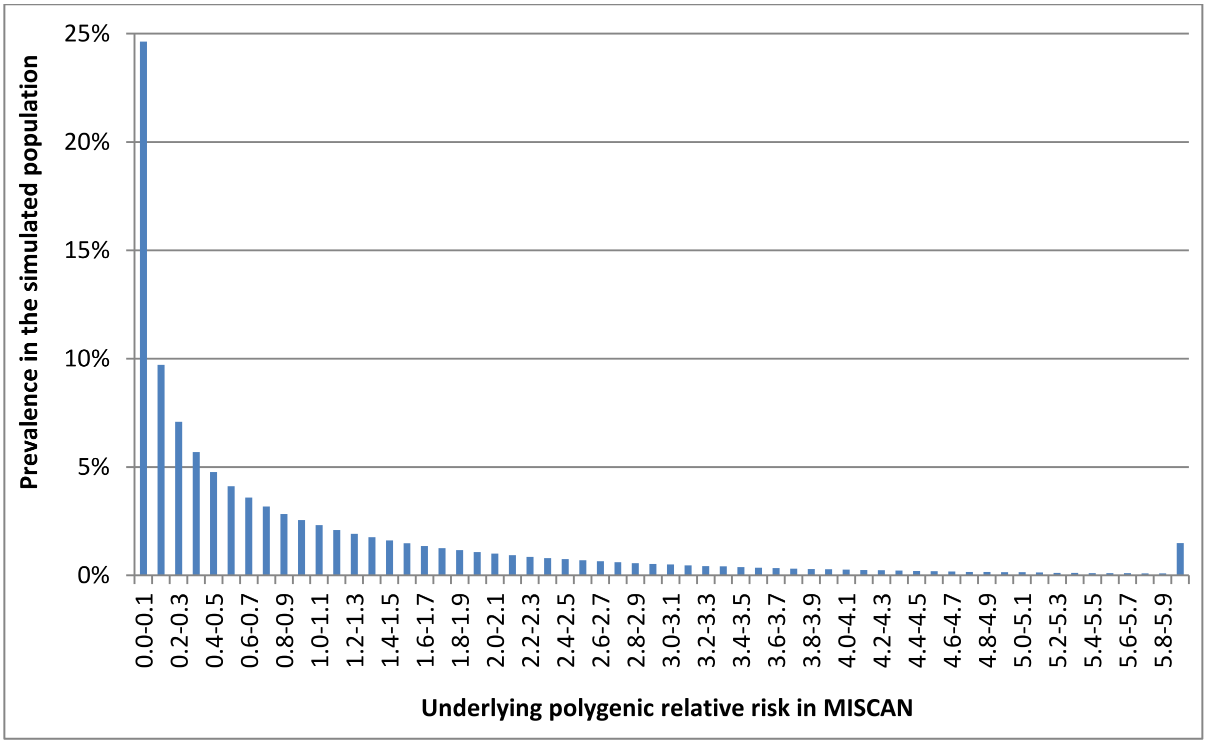

Technical Appendix Figure 2. **Subdivision of the population by estimated polygenic risk, as obtained by polygenic tests with AUC values of 0.60 (i.e., current discriminatory performance), 0.65, …, 0.80 (i.e., potential future discriminatory performance).**

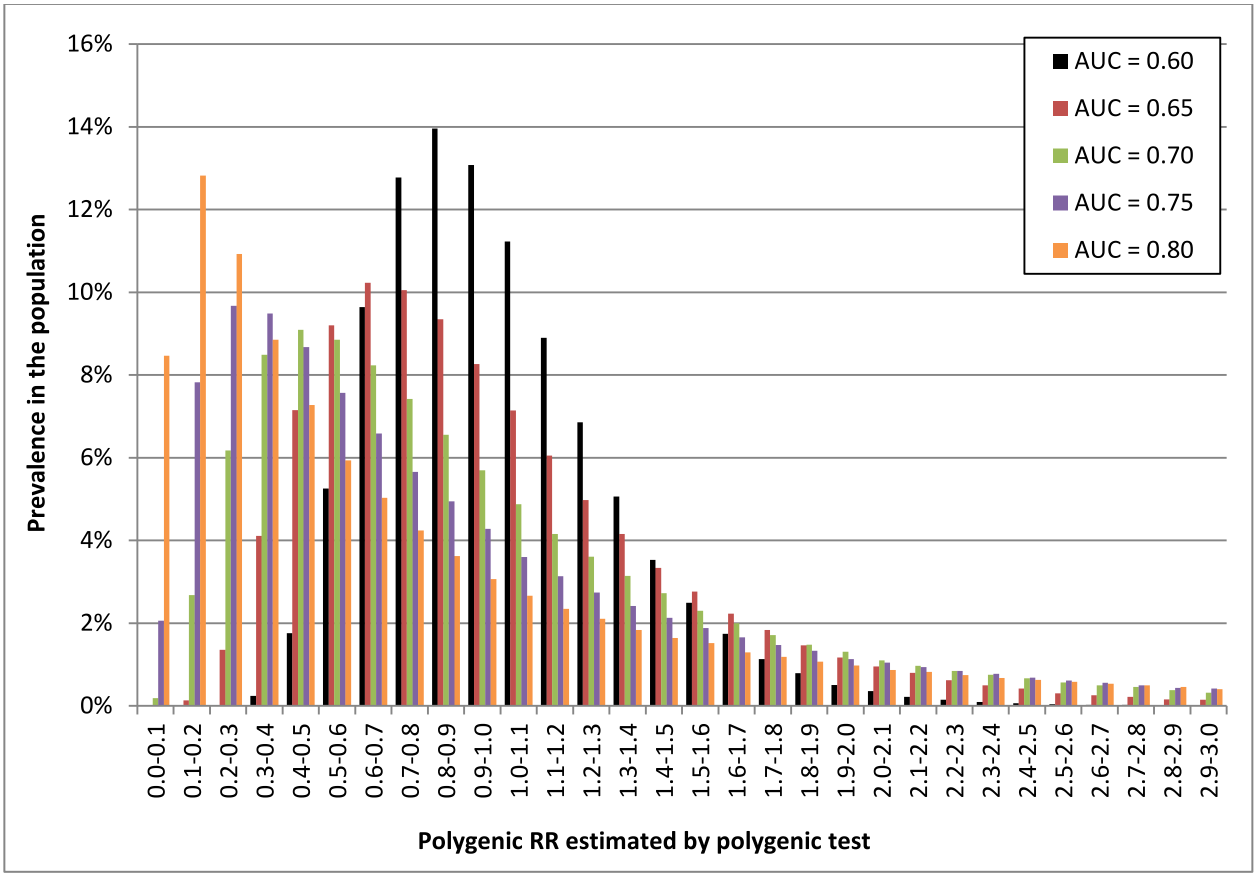

Technical Appendix Figure 3. **Distribution of individuals with an estimated polygenic RR of 0.7-0.8, as estimated by a polygenic test with AUC = 0.60, among polygenic relative risk cohorts in MISCAN.**

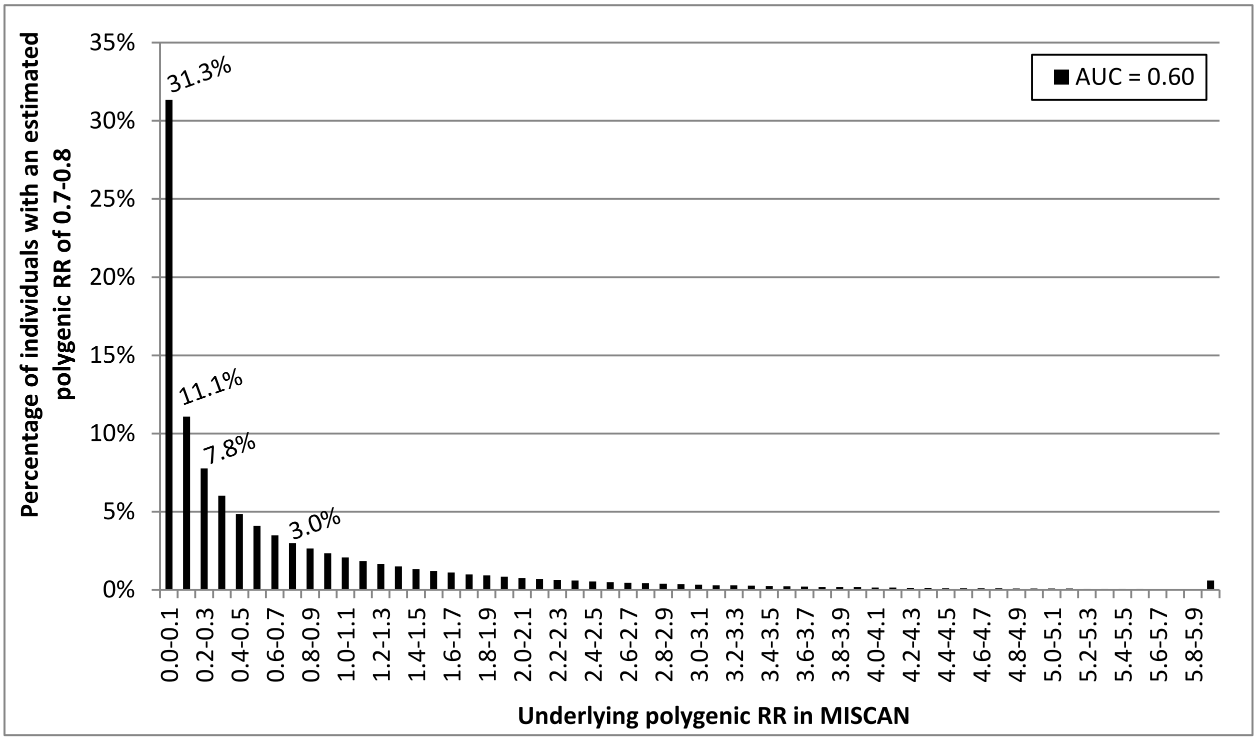

Technical Appendix Figure 4. **Graphical representation of the elliptical copula assuming a polygenic test with AUC = 0.60. The scatterplot shows the concordance between the underlying polygenic RR in MISCAN (x-axis) and the polygenic RR estimated by the polygenic test (y-axis) for 10,000 simulated individuals.**

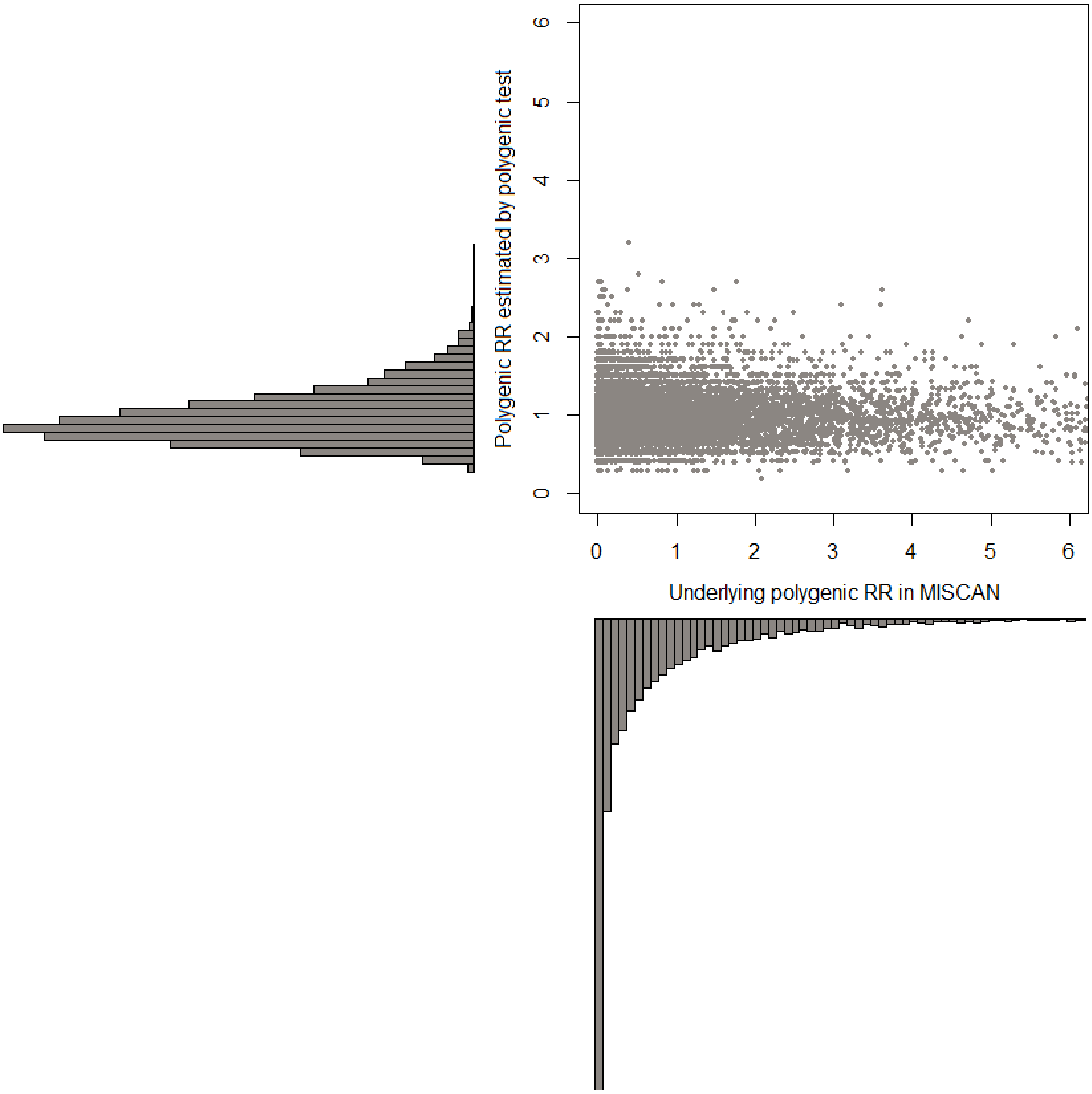

Supplement: pkz086_Supplementary_Data [file pkz086_supplementary_data.pdf]
